# Hydrodynamic Fluidic Pump Empowered Sensitive Recognition and Active Transport of Hydrogen Peroxide in 1D Channels

**DOI:** 10.1002/advs.202408755

**Published:** 2024-11-11

**Authors:** Shuya Liu, Yongxian Guo, Yanjun Gong, Yanze Wei, Qiongzheng Hu, Li Yu

**Affiliations:** ^1^ Key Laboratory of Colloid and Interface Chemistry Ministry of Education Shandong University Jinan 250100 China; ^2^ Qilu University of Technology (Shandong Academy of Sciences) Shandong Analysis and Test Center Jinan 250014 China; ^3^ State Key Laboratory of Biochemical Engineering Institute of Process Engineering Chinese Academy of Sciences Beijing 100190 China

**Keywords:** 1D channel, fluidic pump, mass transport, metal–organic framework, selective trapping

## Abstract

Through synthetic chemistry, the development of molecular devices for the precise selective recognition and active transport of small molecules stands as one of the most ambitious objectives in extensive medical, environmental, and biological applications. The periodical channels of the metal–organic frameworks (MOFs) with excellent chemical affinity offer vast regulatory space for reaching this goal. Herein, by post‐modifying fluorescent probes and ionic liquid molecules into the Zr‐MOFs (NU‐1000), a donor–acceptor (D‐A) system within the periodical 1D channels is created to construct a hydrodynamic fluidic pump within the abundant 1D channels. Irradiation with light serves to initiate and direct fluid motion, expediting the transport of H_2_O_2_ molecules to the active site, thus boosting the sensor sensitivity through gas enrichment. The rapid mass transfer, characterized by a high flow rate and intensified interaction between the D‐A system and H_2_O_2_ molecules, enables the detection of H_2_O_2_ at concentrations as low as 20 ppb. Besides, with the aid of incident light, the pump system exhibits active transport characteristics by transporting radicals derived from H_2_O_2_ against a concentration gradient, reaching a remarkable 10^th^ cycle. The strategy of achieving active transport of small molecules through pore modification holds promise for advancing the development of artificial bioactive channels.

## Graphical abstract

An artificial channel within metal–organic frameworks (NU‐1000), empowered by a fluidic pump, is created through the post‐modification of fluorescent probes and ionic liquid molecules. This engineered system enables highly sensitive detection of H_2_O_2_ within the periodic 1D channels, facilitating the active transport of associated radicals, thus presenting novel possibilities for sensing, trapping, and transporting H_2_O_2_ in bioactive applications.

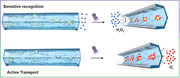

## 1 Introduction

The functions of natural carriers, ion channels, and aquaporins facilitate the selective and highly efficient communication of cells with the external environment, enabling the dynamic maintenance of the cell's osmotic pressure balance with an ultra‐energy‐saving feature.^[^ ^1^, ^2^, ^3^ ^]^ Replicating the intricate natural transporters that regulate transmembrane transport, with their remarkable functional efficiency, to broaden their utility for extracellular use could significantly drive progress in cargo transport,^[^ ^4^ ^]^ mixture separation,^[^ ^5^ ^]^ catalysis,^[^ ^6^ ^]^ chemical sensing,^[^ ^7^ ^]^ and therapy.^[^ ^8^ ^]^ A crucial step toward achieving this objective involves substituting natural transporters with simple yet robust synthetic counterparts: these analogs can mimic biological functions effectively while prioritizing procedural and structural simplicity. Consequently, searching for a suitable matrix amenable to flexible chemical modification strategies has never stopped.

Integrating functional molecules into metal–organic frameworks (MOFs) through covalent bonding, electrostatic interaction, or physical adsorption has proven to be a powerful method for modulating pore structure and chemical environments within MOFs.^[^ ^9^, ^10^, ^11^ ^]^ While successful applications in bioimaging,^[^ ^12^ ^]^ catalysis,^[^ ^13^, ^14^ ^]^ chiral separation,^[^ ^15^, ^16^, ^17^ ^]^ sensing,^[^ ^18^, ^19^, ^20^, ^21^ ^]^ and drug delivery^[^ ^22^, ^23^, ^24^ ^]^ have been explored, a promising yet uncharted territory lies in the incorporation of bio‐active molecules into MOFs for the emulation of biological channels. In theory, by leveraging biological molecules to shape and functionalize the pores of crystalline MOFs, an environment akin to biological channels could be engineered. This concept mirrors achievements in other solid‐state nanopores. For example, the impressive artificial water channels utilizing hydroxyl channels in MOFs, which mimic aquaporins, demonstrate rapid water permeability, precise ion rejection, and feature highly energy‐efficient and robust characteristics.^[^ ^25^ ^]^ This has sparked significant interest in their potential application in commercial water purification processes such as desalination. Apart from water molecules and ions, the precise recognition and active transport of specific small molecules, like H_2_O_2_, are crucial in biological applications, especially in disease diagnosis and treatment.^[^ ^26^, ^27^ ^]^ It is noteworthy that H_2_O_2_ gas molecules serve as a dependable and highly specific indicator. They not only act as an early marker for lung diseases such as asthma and chronic obstructive pulmonary disease but also function as a distinct indicator for high‐risk explosives like triacetone triperoxide.^[^ ^28^, ^29^ ^]^ In practical scenarios, swift and accurate detection is often essential in exhaled breath or intricate gas sampling environments, necessitating the utmost sensitivity in capturing and detecting H_2_O_2_.^[^ ^30^ ^]^ However, challenges arise due to their small size and resistance to external stimuli, hindering their recognition and transportation. Natural organisms typically recognize and transport H_2_O_2_ through specific enzyme systems, e.g., aquaporins bearing phosphorylated sites. The pivotal factor in achieving selective recognition, adsorption, and transportation of H_2_O_2_ within MOFs lies in integrating a precisely tailored active site into the matrix, coupled with the capacity for high‐throughput sieving of transformed molecules. In pursuit of this objective, employing hydrodynamic trapping facilitated by an efficient fluidic pump within meticulously engineered MOFs channels featuring intricately adorned recognition sites emerges as a promising strategy. This approach leverages the controlled fluid flow to effectively immobilize or concentrate H_2_O_2_ molecules within a defined region, thus prolonging the trapping of H_2_O_2_ in complex aqueous solutions, and facilitating their isolation and transportation. In previous explorations, Franzl et al. achieved the trapping and manipulation of nano‐objects near a gold‐liquid interface by triggering thermo‐osmotic flows and optically modulating van der Waals and double‐layer interactions.^[^ ^31^ ^]^ Keumrai et al. reported a single plasmonic cavity membrane that, taking advantage of both capillarity and hydrodynamic trapping, not only enriches and captures a few nucleic acids but also quickly amplifies them for sensitive plasmonic detection.^[^ ^32^ ^]^ With its capacity to rapidly transport and capture minute gaseous molecules at recognition sites within a directional fluid medium, there exists significant potential for enhancing the micro‐environmental design of MOF channels.

**Scheme 1:**
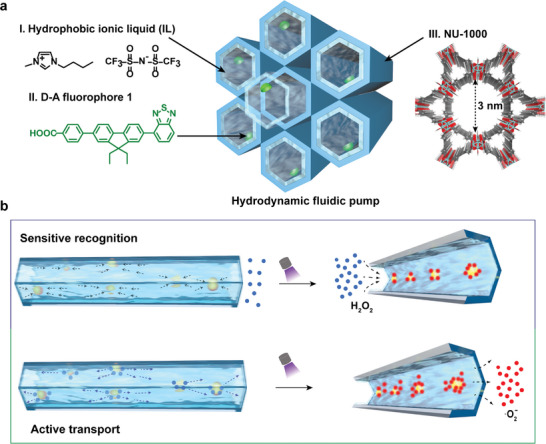
NU‐1000‐**1**/IL composites as the fluidic pump to immobilize and concentrate H_2_O_2_. a) view of NU‐1000‐**1**/IL composites. ILs have been infiltrated in 1D channels of the NU‐1000, surrounding the D‐A fluorophore **1**. b) Schematic illustration of sensitive recognition gaseous H_2_O_2_ molecules and active transport H_2_O_2_‐derived free radicals by the fluidic pump.

In light of the natural carriers, we introduce a concept for an effective fluidic pump‐assisted 1D artificial H_2_O_2_ channels within MOFs for sensitive recognition and active light‐driven transport of corresponding superoxide radicals. To achieve this, a bio‐active donor–acceptor (D–A) molecule, comprising benzothiadiazole and 9,9‐dihexyl fluorene units known for their high luminosity, is introduced into the channels of a Zr‐based MOF (NU‐1000) using a solvent‐assisted ligand incorporation (SALI) method.^[^ ^33^, ^34^ ^]^ Subsequently, ionic liquids (ILs) are decorated onto the NU‐1000 channels through weak interactions to create stable dispersions via physical blending (**Scheme** **1**). The bioactive D‐A molecules within the channels provide numerous recognition sites and generate heat gradients upon exposure to light. When combined with the dynamic fluid environment facilitated by ILs, this setup enables directional fluidic motion within the 1D channels of NU‐1000. This fluidic pump in the 1D artificial channels effectively enriches, interacts with, and adsorbs gaseous molecules through hydrodynamic effects, showcasing a superior sensitivity of gaseous H_2_O_2_ molecules reaches as low as 20 ppb with an excellent selectivity due to the hydrophobic properties of the imidazolyl ILs. Significantly, utilizing D‐A molecules in the construction of the fluidic pump enables the reverse concentration transport of H_2_O_2_‐derived free radicals, leading to a sustained increase in their concentration within the target system across 10 cycles. The sensitive recognition and efficient transport capabilities of the artificial channels within MOFs offer a novel approach for precisely targeting the elimination of cancer cells and for sustained antimicrobial applications.

## 2 Results and Discussion

### 2.1 Synthesis and Characterization of the Fluidic Pump in 1D Channels

To confer sensitive H_2_O_2_ recognition capabilities upon the MOFs channel, a benzothiadiazole‐based D‐A fluorophore **1** was synthesized through Pd‐catalyzed Suzuki cross‐coupling reactions (detailed methods provided in the Supporting Information and Figures S1–S3, Supporting Information). When exposed to light, D‐A fluorophore **1** initiates a charge‐separated state marked by robust electrostatic interactions, efficiently trapping the target H_2_O_2_. This synthesized fluorophore was then integrated into NU‐1000 to establish the recognition sites in the fluidic pump channel using the SALI protocol (see Figures S4 and S5, Supporting Information). In particular, the coordination sites within the Zr‐metal cluster, originally occupied by terminal OH and OH_2_ ligands (Figure S6, Supporting Information),^[^ ^35^ ^]^ establish interactions with the carboxylic acid groups present in the D‐A fluorophore **1**. This interaction guarantees the embedding of the fluorophore within the channels of NU‐1000, ensuring a close and stable integration.

**Figure 1:**
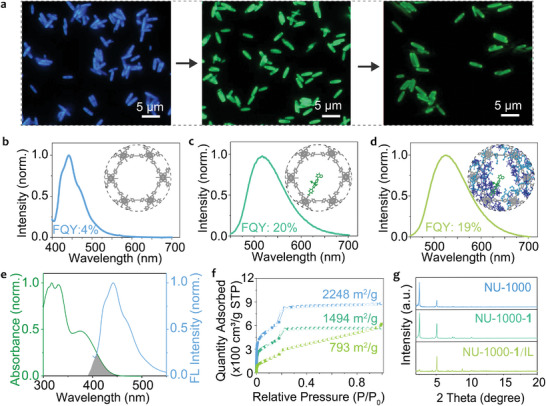
Characterization of NU‐1000, NU‐1000‐**1**, and NU‐1000‐**1**/IL composites. a) Fluorescence microscopy images of NU‐1000 (left), NU‐1000‐**1** (middle), and NU‐1000‐**1**/IL composites (right). The fluorescence spectra and fluorescence quantum yields (FQYs) of NU‐1000 b), NU‐1000‐**1** c), and NU‐1000‐**1**/IL composites d), respectively. e) Normalized absorption spectrum of D‐A fluorophore **1** (green line) and fluorescence spectrum of NU‐1000 (blue line). f) N_2_ adsorption–desorption isotherms of NU‐1000 (blue line), NU‐1000‐**1** (green line), and NU‐1000‐**1**/IL composites (yellow‐green line), respectively. g) The experimental PXRD patterns of NU‐1000, NU‐1000‐**1**, and NU‐1000‐**1**/IL composites.

As depicted in **Figure** **1**a, following the post‐modification with D‐A fluorophore **1**, the emission of NU‐1000 shifts from blue to green. This transition implies a Förster resonance energy transfer (FRET) process occurring from NU‐1000 to D‐A fluorophore **1**, a phenomenon supported by the spectral overlaps between the emission of NU‐1000 and the absorption of D‐A fluorophore **1** (Figure 1e). Furthermore, fluorescence spectra exhibit a red shift from 440 to 517 nm, and the fluorescence quantum yield (FQY) also increased from 4% to 20% (Figures 1b,c). The successful formation of NU‐1000‐**1** composites was further validated through Fourier‐transform infrared (FT‐IR) experiments (Figure S7, Supporting Information). The observed shift in the ν(C═O) stretching band, from 1684 to 1703 cm^−1^, indicates the formation of a coordination bond between the carbonyl group of D‐A fluorophore **1** and the Zr cluster in NU‐1000, caused a shift in the position of the carbonyl stretching vibration of D‐A fluorophore **1**. Moreover, the EDS line‐scan profile demonstrates a uniform distribution of D‐A fluorophore **1** within NU‐1000‐**1** (Figure S8, Supporting Information). Inductively coupled plasma mass spectrometry (ICP‐MS) analysis, as presented in Table S1 (Supporting Information), confirms that the molar ratio of the loaded D‐A fluorophore **1** with a sulfur atom to Zr‐cluster was 1:1. This suggests an average functionalization of nearly three D‐A fluorophore **1** molecules for each hexagonal pore (Figure S9, Supporting Information).

Subsequently, hydrophobic imidazolium‐based ILs ([C_4_mim][NTf_2_]) were introduced into the channels of NU‐1000 through physical blending, leading to the creation of stable NU‐1000‐**1**/IL composites facilitated by electrostatic interactions. The full configuration of the fluidic pump with hydrodynamic trapping leverages the directional fluid flow to immobilize and concentrate H_2_O_2_ within specific channel regions of NU‐1000‐**1**/IL composites. The zeta potential of NU‐1000‐**1** and NU‐1000‐**1**/IL shifted from −3.53 to −0.56 mV as depicted in Figure S10 (Supporting Information), indicating a significant change in the surface charge of the material. UV–Visible‐NIR microscope and spatially resolved fluorescence spectra of NU‐1000‐**1**/IL (Figure S11, Supporting Information) show the fluorescence emission peaks at different locations on a single NU‐1000‐**1**/IL crystal are consistent with the maximum emission wavelength at 525 nm, demonstrating IL almost evenly distributed in the channels of NU‐1000‐**1**. The emissions from these composites experienced a slight redshift and the FQY also dropped a bit (Figures 1c,d), attributed to the solvation effect of the polar solvent (Figure S12, Supporting Information).^[^ ^36^ ^]^ Time‐resolved emission spectra were also used to demonstrate IL has been infiltrated into the channels of NU‐1000. As shown in Figure S13 and Table S2 (Supporting Information), D‐A fluorophore **1** in IL shows an exponential decay with an average lifetime of 6.92 ns. NU‐1000‐**1** shows an exponential decay with an average lifetime of 4.26 ns, while NU‐1000‐**1**/IL shows a faster exponential decay with an average lifetime of 2.56 ns. This faster decay is because the polarity environment of the D‐A fluorophore **1** is changed by the IL, which increases the transition pathways of the excited states. Besides, the NU‐1000‐**1**/IL and NU‐1000‐**1** were also analyzed using the scanning TEM (STEM) coupled with energy dispersive X‐ray spectroscopy (EDX). The analysis focuses on the elemental distribution and content of C, Zr, S, and F species, respectively. Figure S14 (Supporting Information) gives evidence of the uniform distribution of C, Zr, S, and F species over the entire NU‐1000, demonstrating the presence of ionic liquids across the entire surface of the 1D channels in the NU‐1000. As shown in Figure S14b,c (Supporting Information), a high and homogenous distribution of F (≈17.5 at%) and S signals (≈11.1 at%) can also be confirmed the ILs in the NU‐1000‐**1**/IL, whereas the NU‐1000‐**1** without ionic liquid has a very low distribution of the F signal (≈0.40 at%) and the S signal (≈1.33 at%) in Figure S15 (Supporting Information). These results demonstrate that the ionic liquid has infiltrated into the channels of NU‐1000 to form NU‐1000‐**1**/IL. To assess alterations in the channels of the NU‐1000 frameworks post‐modification with D‐A fluorophore **1** molecule and ILs, we analyzed the N_2_ adsorption–desorption isotherms. Figure 1f displays the Brunauer–Emmett–Teller (BET) results, indicating a decrease in surface area from 2248 to 1494 m^2^·g^−1^, and further to 793 m^2^·g^−1^ upon the introduction of D‐A fluorophore **1** molecules and ILs, respectively. Notably, the post‐modified samples exhibited the Type IV isotherm shape characteristic of pristine NU‐1000, suggesting the preservation of its mesoporous characteristic during post‐modification, which is demonstrated by the pore size distributions of NU‐1000‐**1/**IL composites (Figure S16, Supporting Information). This finding aligns with the powder X‐ray diffraction (PXRD) data (Figure 1g and Figure S17, Supporting Information), which displayed consistent structural patterns before and after the post‐modification of NU‐1000, indicating good crystallinity. The decrease of the 2.54° (100) peak for NU‐1000‐**1/**IL composites is mainly due to the crystal orientation which aligned parallel to the substrate^[^ ^37^ ^]^ (Figure S18, Supporting Information).

**Figure 2:**
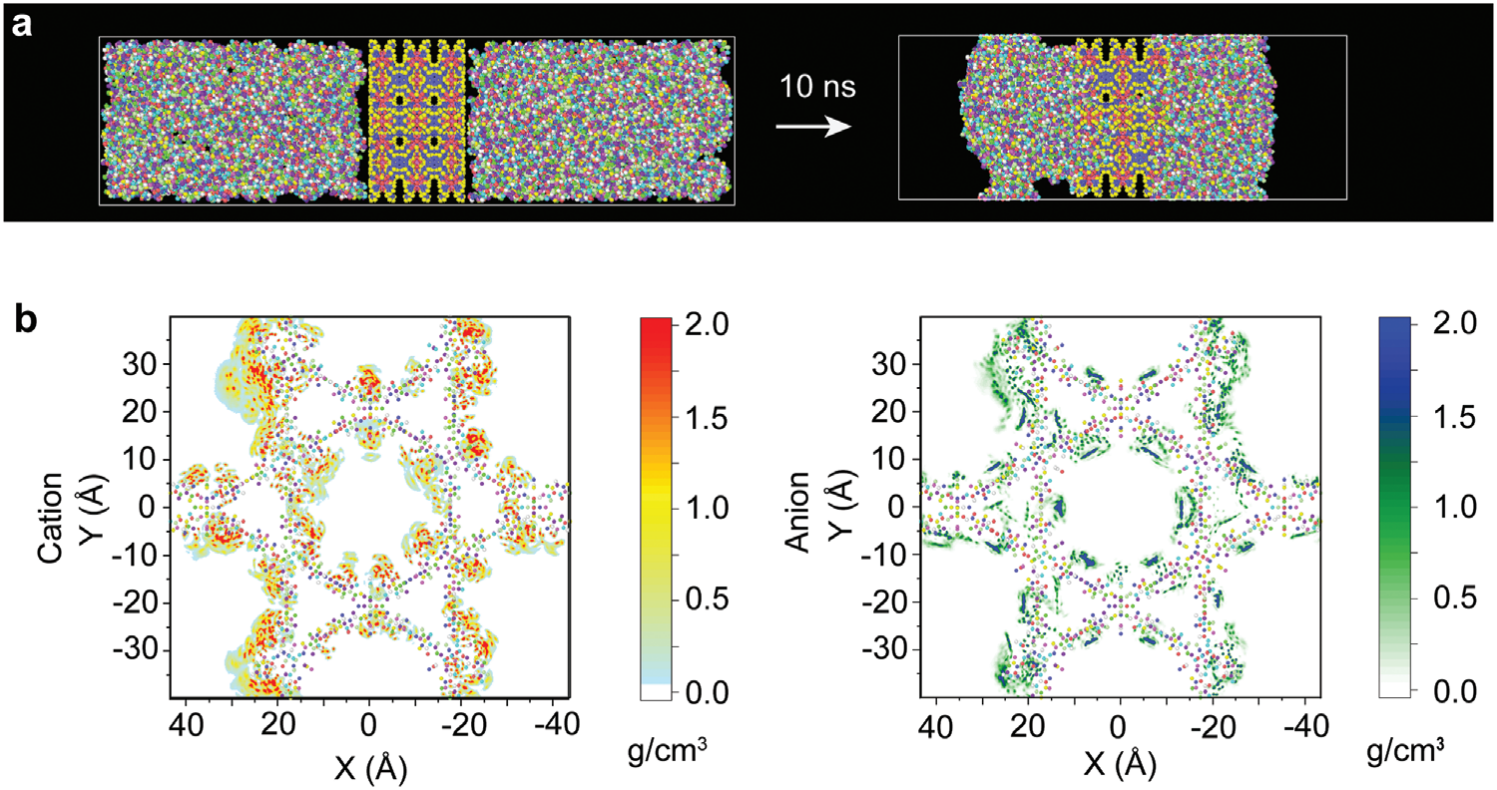
Molecular simulations of NU‐1000/IL composites. a) Snapshots from 10 ns MD simulations of infiltration into NU‐1000 for ILs at 300 K. b) 2D maps of ion distributions of [C_4_mim] cation (left) and [NTf_2_] anion (right) inside channels of NU‐1000.

The state and spatial arrangement of ILs within the channels is crucial for comprehending the hydrodynamic fluidic pump system, enabling selective mass transfer and sensitive detection of H_2_O_2_. Through molecular dynamics (MD) simulations employing atomistic models of MOFs^[^ ^38^ ^]^ and ILs,^[^ ^39^ ^]^ the movement of IL molecules within the channels of NU‐1000‐**1**/IL composites is unveiled. The simulation outcomes vividly illustrate the kinetics of ILs infiltrating NU‐1000, as evidenced in time‐series plots delineating the movement of IL molecules along the *c*‐axis. Notably, these molecules swiftly permeated the pores of NU‐1000 (**Figure** **2**a and Movies S1 and S2, Supporting Information), predominantly congregating on the surface of the hexagonal pores (3 nm in diameter) (Figure 2b). This phenomenon is likely attributed to the electrically charged Zr clusters in NU‐1000 pores, which attracted ILs to their surfaces through electrostatic interactions. Upon reaching molecular equilibrium in MD simulations, 2D in‐plane maps depicted the charge and ion distributions of ILs within NU‐1000 channels (Figure 2b). These molecules exhibited a consistent distribution, forming a liquid phase layer on the channel surfaces of NU‐1000. Notably, ions were unevenly dispersed around these channels, with cations situated near Zr clusters and anions in proximity to organic linkers. This non‐uniform distribution could enhance the catalytic characteristics of NU‐1000. Therefore, these NU‐1000/IL composites have the potential to function as efficient fluidic pumps, facilitating precise fluid control.

### 2.2 Mass Transport in Fluidic Pump System

**Figure 3:**
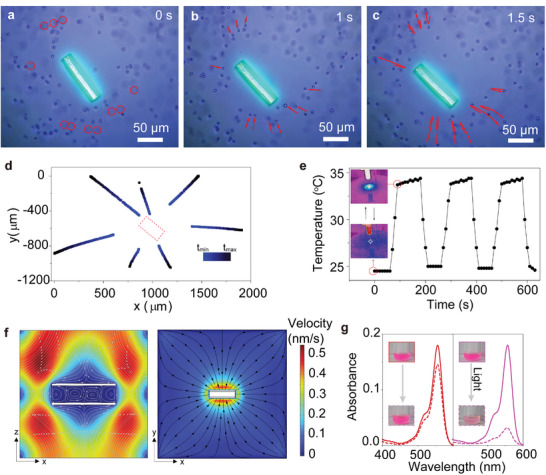
Hydrodynamic trapping under light irradiation and heat convection flow field. a–c) Snapshots of the fluid trapping process by NU‐1000‐**1**/IL under 330–390 nm light. d) Directional particle trajectories of 4 µm diameter PS microspheres to trace the trajectory change of fluid motion. e) Photothermal conversion of NU‐1000‐**1**/IL composites during heating and cooling in three cycles. Inset: Photothermal images of NU‐1000‐**1**/IL with and without exposure to the 365 nm light. f) The motion rate of the ILs with the temperature rise of NU‐1000‐**1**/IL simulated by fluid dynamics. g) UV–vis spectra of 0.1 mg mL^−1^ Rhodamine B in IL solutions using 1 mg NU‐1000‐**1**/IL with and without UV irradiation.

Typically, enhanced fluid flux, coupled with the selective enrichment and capture of specific molecules, forms the cornerstone for achieving rapid screening. In NU‐1000‐**1**/IL composites, the fluidic pump system was meticulously engineered to ensure precisely controlled flow dynamics through hydrodynamic trapping, resulting in heightened target capture by D‐A fluorophore **1** upon illumination. This capturing capability was facilitated by the strategic introduction of ILs into the channels of NU‐1000‐**1** via the pump, enabling swift target enrichment. To visually track fluid trajectories in real‐time using fluorescence‐mode microscopy, we employed monodisperse polystyrene (PS) microspheres with a diameter of 4 µm. The observed fluid trapping process was illustrated in **Figure** **3**a–d, along with Movies S3 and S4 (Supporting Information). When exposed to light, the PS particles migrated toward NU‐1000‐**1** and then reversed direction. This phenomenon underscores the efficient fluid circulation induced by hydrodynamic trapping, potentially amplifying detection sensitivity through target enrichment. The driving force behind the fluidic pump's operation stemmed from the temperature gradient initiated by the heat generated from D‐A fluorophore **1** within the channels of NU‐1000 upon light exposure. The directional movement of the fluid was governed by the confined dimensions of the 1D channels of NU‐1000, culminating in a fluid vortex. The resulting temperature gradient was visualized using a thermal imager. As demonstrated in Figure 3e, Figure S19 and Movie S5 (Supporting Information), the temperature of NU‐1000‐**1**/IL composites remained stable at 25 °C in the absence of light exposure. However, upon UV light exposure (365 nm), the temperature rapidly rose to 34 °C within 20 s. The temperature of the fluidic pump system remained constant at 34 °C under continuous light exposure and promptly reverted to its initial value upon light removal. While the thermal imaging of NU‐1000/IL (Figure S20, Supporting Information) and only glass (Figure S21, Supporting Information) indicates that the temperature of control samples remained stable ≈22–23 °C during 120 s of UV light exposure. These results clearly demonstrate that the driving force behind the fluidic pump's operation stems from the temperature gradient generated by the heat from D‐A fluorophore **1**, rather than any heat contribution from NU‐1000 itself.

To validate the observed phenomenon of light‐induced directional fluid flow, a 3D fluid dynamics simulation was performed. Focusing on the fluid's directional motion within the 1D pore of NU‐1000, we simplified the complex multichannel structure of NU‐1000 into a single‐channel model. The fluid flow was analyzed using a fluid‐structure interaction (FSI) method, where the incompressible Navier–Stokes equation was employed to calculate fluid dynamics. Figure 3f showcases the fluid behavior as the temperature increases, depicting a direct flow into the channel, forming a vortex near the heat source before redirecting the liquid within the channel. To further demonstrate that the controlled directional fluid flow can enhance target enrichment efficiency, we conducted adsorption experiments of Rhodamine B in ionic liquid (IL) solutions (0.1 mg mL^−1^) using the NU‐1000‐**1**/IL composites with and without UV light exposure (330–390 nm). Figure 3g illustrates that the absorption of Rhodamine B molecules exceeded 80% after 3 h of light exposure, compared to only 18% without light irradiation. This result indicates that light exposure significantly accelerated the capture process of the dye molecules, underscoring the effectiveness of controlled directional fluid flow in expediting target enrichment.

### 2.3 Selective Recognition and Active Transport of H_2_O_2_ Molecules

**Figure 4:**
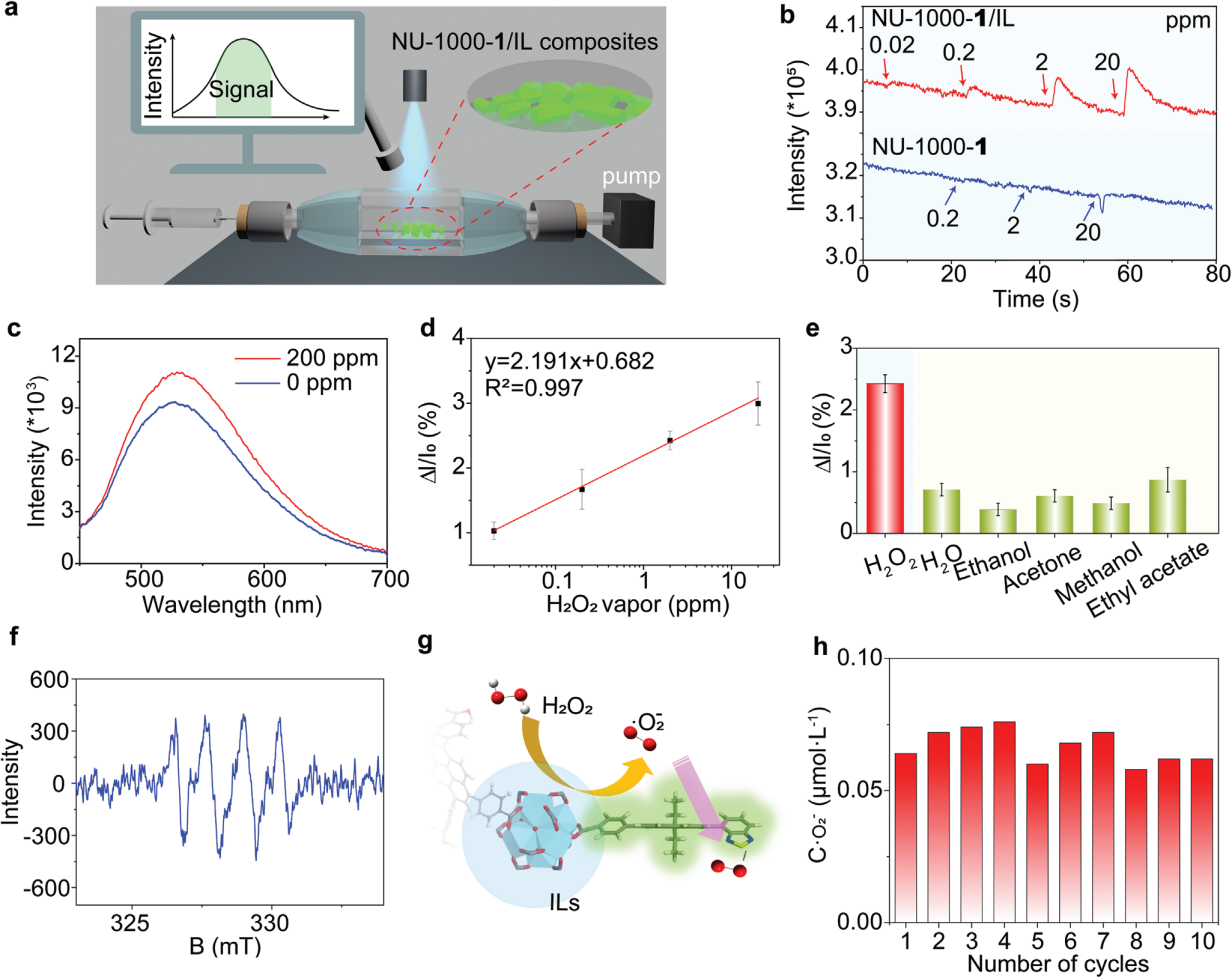
Selective recognition and active transport of H_2_O_2_. a) The experimental setup consists of a transparent sample chamber, an LED light source with a wavelength of λ = 385 nm, an external pump, and a fiber optical spectrometer, the response signal obtained by the changes in the peak area of fluorescence emission. b) Time‐dependent fluorescence profiles of NU‐1000‐**1**/IL composites and NU‐1000‐**1** post‐modification in 5 mg mL^−1^ D–A fluorophore **1** upon exposure to H_2_O_2_ vapor at different concentrations. c) Enhanced fluorescence spectra of NU‐1000‐**1**/IL in the presence and absence of 200 ppm H_2_O_2_ under UV light (385 nm, 0.58 mW cm^−2^) irradiation. d) Experimental and fitted fluorescence enhancement ratios of NU‐1000‐**1**/IL composites upon exposure to H_2_O_2_ vapor at different concentrations. Error bars represent the standard deviation of five measurements. e) Columnar comparison of fluorescence responses of NU‐1000‐**1**/IL composites upon exposure to 2 ppm H_2_O_2_ vapor and various potential interferents (H_2_O: 3100 ppm; Ethanol: 500 ppm; Acetone: 240 ppm; Methanol: 1300 ppm; Ethyl acetate: 1000 ppm). ΔI/I_0_ represents the change in fluorescence intensity. Error bars represent the standard deviation of five measurements. f) ESR spectra of 2 mg NU‐1000, 20 µL of DMPO, 100 µL [C_4_mim][NTf_2_], and 0.5 mL of 100 mM H_2_O_2_. g) A schematic diagram of the fluorescence enhancement signal generated by the interaction of fluorophore **1** with superoxide anion radicals produced by H_2_O_2_ through Zr‐clusters of NU‐1000 catalyzed under the Lewis acidic condition provided by ILs. h) The release content of superoxide anion radicals at different cycle numbers when NU‐1000‐**1**/IL captured H_2_O_2._

Subsequently, we conducted a thorough investigation into the capture and detection capabilities of NU‐1000‐**1**/IL for H_2_O_2_ vapor. We validated the influence of the D‐A system and fluidic pump system on the detection limit and the efficiency of H_2_O_2_ molecule enrichment through comparative experiments.^[^ ^28^, ^29^, ^30^ ^]^ The selective detection of H_2_O_2_ was confirmed using a sensing platform capable of detecting this target molecule even in complex environments, which allows the detection of gaseous H_2_O_2_ in high‐humidity conditions. Using a custom‐built optical chamber equipped with an Ocean Optics fluorometer, we investigated the sensing performance, with the results depicted in **Figure** **4**a. By optimizing the post‐modification content of various D‐A fluorophores, it was observed that the fluidic pump system with 5 mg/mL D‐A fluorophore **1** exhibited increased sensitivity and excellent photostability when detecting H_2_O_2_ vapor at elevated humidity levels, as illustrated in Figure 4b,c and Figure S22 (Supporting Information). We notice that the presence of water vapor did not significantly affect the fluorescence signal of the fluidic pump system (Figure S23, Supporting Information), indicating the effective mitigation of water vapor interference by the ionic liquid (IL) within NU‐1000‐**1**. Remarkably, the limit of detection for H_2_O_2_ in high‐humidity environments was found to be as low as 20 ppb, as shown in Figure 4b. Furthermore, the fluorescence response was rapid, with a response time of ≈5.0 s (Figure S24, Supporting Information). Figure 4d demonstrates a linear correlation between the fluorescence enhancement ratio (ΔI/I_0_) of the fluidic pump system and varying concentrations of H_2_O_2_ vapor, ranging from 0.02 to 20 ppm. To elucidate the role of hydrodynamic trapping in detection, it is noteworthy that when NU‐1000‐**1** alone was exposed to H_2_O_2_ vapor in high‐humidity conditions, a noticeable quenching of the fluorescence response was observed (as depicted in Figure 4b), similar to its response to water vapor (Figure S25, Supporting Information). This suggests that the fluorescence response was influenced by water vapor and that the detection of trace amounts of H_2_O_2_ vapor without ILs was not achievable. These results highlight that the ultra‐sensitive detection of H_2_O_2_ vapor by the fluidic pump system can be attributed to the hydrodynamic trapping effect of NU‐1000‐**1**/IL under light irradiation. In order to validate the 1D channels structure of the NU‐1000 as a good platform for hydrodynamic trapping effects to be used for enhanced detection of H_2_O_2_ vapor, we conducted additional control experiments using MOF‐808 and PCN‐777. MOF‐808‐**1**/IL and PCN‐777‐**1**/IL composites were synthesized by introducing D‐A fluorophore **1** and ionic liquid [C_4_mim][NTf_2_] into MOF‐808 and PCN‐777 sequentially under the same conditions as those used to prepare NU‐1000‐**1**/IL. The structural integrity (Figures S27,S28, Supporting Information), morphology (Figures S29,S30, Supporting Information) and fluorescence properties (Figure S31, Supporting Information) of the post‐synthetic modification MOFs were analyzed. Due to the small three channel size of MOF‐808 and the non‐1D channels of PCN‐777 (Figure S26, Supporting Information), which lack hydrodynamic trapping effect, we observed no enhancement in fluorescence signals upon exposure to H₂O₂ vapor (Figure S32, Supporting Information). These results demonstrated the NU‐1000 can serve as a suitable platform for enhanced fluorescence sensing sensitivity with hydrodynamic trapping effects due to its periodic mesoporous 1D channels.

Moreover, the fluidic pump system demonstrates exceptional selectivity for H_2_O_2_ vapor over other potential interfering agents, as evidenced in Figure 4e and Figure S33 (Supporting Information). Exposure of the fluidic pump system to various volatile organic compounds resulted in minimal fluorescence responses compared to the significant fluorescence enhancement induced by H_2_O_2_ vapor (2 ppm). Compared to other detection methods, the fluidic pump system has a faster response time, a wider linear range, and higher sensitivity for H_2_O_2_ sensing (Table S3, Supporting Information). Then, our hypothesis posited that the selective adsorption of H_2_O_2_ vapor stemmed from the catalytic dissociation of H_2_O_2_ into superoxide anion (•O_2_^−^), facilitated by the synergistic interplay between NU‐1000 and the IL. To further elucidate this hypothesis, the electron spin resonance (ESR) spin‐trap method was employed to investigate the reactive oxygen species using 5,5‐dimethyl‐1‐pyrroline N‐oxide (DMPO) as traps. The distinct characteristic four peaks of DMPO‐•O_2_^−^ signals, with relative intensities of 1:1:1:1, were observed in the mixture of NU‐1000, H_2_O_2_, [C_4_mim][NTf_2_], and DMPO, confirming the generation of •O_2_^−^ radicals (Figure 4f). This finding was reinforced by experimental data from the color reaction of 3,3'',5,5″‐tetramethylbenzidine (TMB) and •O_2_^−^ radicals. The absorption value at 430 nm exhibited a notable increase in the mixture of NU‐1000‐**1**, H_2_O_2_, [C_4_mim][NTf_2_], and TMB. Conversely, upon the addition of superoxide dismutase (SOD), no increase was observed, indicating the presence of •O_2_^−^ radicals (Figure S34, Supporting Information). Through the hydrodynamic trapping of H_2_O_2_ within NU‐1000′s channels, the catalytic oxidation of H_2_O_2_ to superoxide anion was accelerated by the Zr clusters of NU‐1000 in the weakly acidic environment of the imidazolium cation. Subsequently, with the directional fluid movement, the •O_2_^−^ radicals were transported to interact with fluorophore **1** (Figure 4g). This interaction disrupted the solvation effect of the IL on the D‐A molecules, leading to an amplified fluorescence signal.

Next, the capacity of NU‐1000‐**1**/IL to actively transport superoxide anions was explored by utilizing the hydroxylamine hydrochloride oxidation method to quantify the superoxide anion levels (refer to the standard curve in Figure S35, Supporting Information). Initially, we utilized NU‐1000‐**1**/IL to capture H_2_O_2_, followed by re‐dispersing the H_2_O_2_‐loaded NU‐1000‐**1**/IL in an aqueous solution containing the original H_2_O_2_ concentration to evaluate its capability to release superoxide anions. When exposed to 365 nm UV light irradiation, NU‐1000‐**1**/IL effectively releases superoxide radicals by leveraging an inverse chemical concentration gradient, showcasing its active superoxide radical transport ability. Additionally, the energy source was verified to be UV light, as the release of superoxide anions was three times higher under irradiated conditions compared to non‐irradiated scenarios. (Figure S36 and Table S4, Supporting Information), demonstrating that illumination accelerated the release of captured H_2_O_2_ in the form of superoxide anions. Furthermore, we examined the cyclic capture and release performance of the NU‐1000‐**1**/IL, as shown in Figure 4h, after 10 cycles, the NU‐1000‐**1**/IL was still capable of releasing stored H_2_O_2_ as superoxide anions, with a release concentration ranging from 0.06 to 0.08 µmol·L^−1^.

## 3 Conclusion

In summary, we have demonstrated an efficient fluidic pump system to enrich target molecules using the hydrodynamic trapping principle triggered by light irradiation for highly sensitive detection of H_2_O_2_ within the periodic 1D channels and active transport of associated radicals to against the concentration gradient. Experiments and molecular dynamics simulations demonstrate that ILs adhere to the large channels of NU‐1000 via electrostatic interactions to form a continuous fluidic layer. The thermal gradient induced by light, stemming from non‐radiative transitions in fluorophore **1**, powers the fluidic pump system, which rapidly enriches target molecules, guiding H_2_O_2_ toward fluorescent probe sites. This process amplifies collision probabilities, significantly elevating detection sensitivity. Demonstrating its efficacy, the efficient pump system showcases the capability to detect trace levels of gaseous H_2_O_2_, as low as 20 ppb, even in high humidity environments. Moreover, leveraging light assistance, the pump system demonstrates active transport of radicals originating from H_2_O_2_, achieving an impressive 10th cycle. In essence, these efficient fluidic pumps pave the way for the advancement of rapid and highly sensitive artificial bioactive channels.

## Conflict of Interest

The authors declare no conflict of interest.

## Supporting information



Supporting Information

Supplemental Movie 1

Supplemental Movie 2

Supplemental Movie 3

Supplemental Movie 4

Supplemental Movie 5

## Data Availability

The data that support the findings of this study are available in the supplementary material of this article.

## References

[1] G. Xue , Y. Xu , T. Ding , J. Li , J. Yin , W. Fei , Y. Cao , J. Yu , L. Yuan , L. Gong , J. Chen , S. Deng , J. Zhou , W. Guo , Nat. Nanotech. 2017, 12, 317. 10.1038/nnano.2016.300 28135262

[2] T. Muraoka , K. Umetsu , K. V. Tabata , T. Hamada , H. Noji , T. Yamashita , K. Kinbara , J. Am. Chem. Soc. 2017, 139, 18016. 29077401 10.1021/jacs.7b09515

[3] W. Ke , K. A. Afonin , Drug Deliv. Rev. 2021, 176, 113835. 10.1016/j.addr.2021.113835 PMC8440450 34144087

[4] L. Zheng , H. Zhao , Y. Han , H. Qian , L. Vukovic , J. Mecinović , P. Král , W. T. S. Huck , Nat. Chem. 2019, 11, 359. 30664718 10.1038/s41557-018-0204-7

[5] X. Li , W. Lin , V. Sharma , R. Gorecki , M. Ghosh , B. A. Moosa , S. Aristizabal , S. Hong , N. M. Khashab , S. P. Nunes , Nat. Commun. 2023, 14, 3112. 37253741 10.1038/s41467-023-38728-7 PMC10229579

[6] M. Wittwer , U. Markel , J. Schiffels , J. Okuda , D. F. Sauer , U. Schwaneberg , Nat. Catal. 2021, 4, 814.

[7] J. M. Kefauver , A. B. Ward , A. Patapoutian , Nature 2020, 587, 567. 33239794 10.1038/s41586-020-2933-1 PMC8477435

[8] Q.‐H. Ling , Y. Fu , Z.‐C. Lou , B. Yue , C. Guo , X. Hu , W. Lu , L. Hu , W. Wang , M. Zhang , H.‐B. Yang , L. Xu , Adv. Sci. 2024, 11, 2308181. 10.1002/advs.202308181 PMC11151027 38459671

[9] S. M. Cohen , Chem. Rev. 2012, 112, 970. 21916418 10.1021/cr200179u

[10] J. Hao , X. Xu , H. Fei , L. Li , B. Yan , Adv. Mater. 2018, 30, 1705634. 10.1002/adma.201705634 29388716

[11] S. Mandal , S. Natarajan , P. Mani , A. Pankajakshan , Adv. Funct. Mater. 2021, 31, 2006291.

[12] H.‐S. Wang , Coord. Chem. Rev. 2017, 349, 139.

[13] W. Xu , Y. Wu , W. Gu , D. Du , Y. Lin , C. Zhu , Chem. Soc. Rev. 2024, 53, 137. 38018371 10.1039/d3cs00767g

[14] W. Chen , P. Cai , H.‐C. Zhou , S. T. Madrahimov , Angew. Chem., Int. Ed. 2024, 63, e202315075. 10.1002/anie.202315075 38135664

[15] Y. Peng , T. Gong , K. Zhang , X. Lin , Y. Liu , J. Jiang , Y. Cui , Nat. Commun. 2014, 5, 4406. 25030529 10.1038/ncomms5406

[16] S. Das , S. Xu , T. Ben , S. Qiu , Angew. Chem., Int. Ed. 2018, 57, 8629. 10.1002/anie.201804383 29770554

[17] T. Chen , H. Li , X. Shi , J. Imbrogno , D. Zhao , J. Am. Chem. Soc. 2024, 146, 14433. 38757701 10.1021/jacs.4c04164

[18] L. E. Kreno , K. Leong , O. K. Farha , M. Allendorf , R. P. Van Duyne , J. T. Hupp , Chem. Rev. 2012, 112, 1105. 22070233 10.1021/cr200324t

[19] L.‐T. Zhang , Y. Zhou , S.‐T. Han , Angew. Chem., Int. Ed. 2021, 60, 15192. 10.1002/anie.202006402 32845072

[20] H. Yuan , N. Li , W. Fan , H. Cai , D. Zhao , Adv. Sci. 2022, 9, 2104374. 10.1002/advs.202104374 PMC8867161 34939370

[21] Q. Qiu , S. Sun , H. Yuan , S. Zhang , Y. Feng , F. Wang , Y. Zhu , M. Zhou , Y. Wang , Biosens. Bioelectron. 2024, 251, 116114. 38354495 10.1016/j.bios.2024.116114

[22] P. Horcajada , C. Serre , M. Vallet‐Regí , M. Sebban , F. Taulelle , G. Férey , Angew. Chem., Int. Ed. 2006, 45, 5974. 10.1002/anie.200601878 16897793

[23] W. Cai , J. Wang , C. Chu , W. Chen , C. Wu , G. Liu , Adv. Sci. 2019, 6, 1801526. 10.1002/advs.201801526 PMC6325578 30643728

[24] Z. Zhou , M. Vázquez‐González , I. Willner , Chem. Soc. Rev. 2021, 50, 4541. 33625421 10.1039/d0cs01030h

[25] N. Hanikel , X. Pei , S. Chheda , H. Lyu , W. Jeong , J. Sauer , L. Gagliardi , O. M. Yaghi , Science 2021, 374, 454. 34672755 10.1126/science.abj0890

[26] J. Chen , L. Chen , Y. Wu , Y. Fang , F. Zeng , S. Wu , Y. Zhao , Nat. Commun. 2021, 12, 6870. 34824274 10.1038/s41467-021-27233-4 PMC8617030

[27] Z. Dong , Z. Yang , Y. Hao , L. Feng , Nanoscale 2019, 11, 16164. 31453999 10.1039/c9nr04418c

[28] C. Johansson , F. C. M. Kirsebom , Mucosal Immunol. 2021, 14, 815. 33758367 10.1038/s41385-021-00397-4 PMC7985581

[29] R. Stolarek , P. Bialasiewicz , M. Krol , D. Nowak , Clin. Chim. Acta 2010, 411, 1849. 20804745 10.1016/j.cca.2010.08.031

[30] X. Yu , Y. Gong , W. Xiong , M. Li , J. Zhao , Y. Che , Anal. Chem. 2019, 91, 6967. 31081320 10.1021/acs.analchem.9b01255

[31] M. Franzl , F. Cichos , Nat. Commun. 2022, 13, 656. 35115502 10.1038/s41467-022-28212-z PMC8813924

[32] K. Whang , J. Min , Y. Shin , I. Hwang , H. Lee , T. Kwak , J. A. La , S. Kim , D. Kim , L. P. Lee , T. Kang , Adv. Mater. 2024, 36, 2403896. 10.1002/adma.202403896 38663435

[33] X. Li , J. Yu , D. J. Gosztola , H. C. Fry , P. Deria , J. Am. Chem. Soc. 2019, 141, 16849. 31566956 10.1021/jacs.9b08078

[34] T. C. Wang , N. A. Vermeulen , I. S. Kim , A. B. Martinson , J. F. Stoddart , J. T. Hupp , O. K. Farha , Nat. Protoc. 2016, 11, 149. 26678084 10.1038/nprot.2016.001

[35] I. Hod , P. Deria , W. Bury , J. E. Mondloch , C. W. Kung , M. So , M. D. Sampson , A. W. Peters , C. P. Kubiak , O. K. Farha , J. T. Hupp , Nat. Commun. 2015, 6, 8304. 26365764 10.1038/ncomms9304 PMC4647847

[36] L. Cui , Y. Gong , C. Cheng , Y. Guo , W. Xiong , H. Ji , L. Jiang , J. Zhao , Y. Che , Adv. Sci. 2021, 8, 2002615. 10.1002/advs.202002615 PMC7887598 33643792

[37] S. Goswami , I. Hod , J. D. Duan , C.‐W. Kung , M. Rimoldi , C. D. Malliakas , R. H. Palmer , O. K. Farha , J. T. Hupp , J. Am. Chem. Soc. 2019, 141, 17696. 31608628 10.1021/jacs.9b07658

[38] P. G. Boyd , S. M. Moosavi , M. Witman , B. Smit , J. Phys. Chem. Lett. 2017, 8, 357. 28008758 10.1021/acs.jpclett.6b02532 PMC5253710

[39] E. K. Watkins , W. L. Jorgensen , J. Phys. Chem. A 2001, 105, 4118.

