# Supplementary material for: Hydrodynamic Fluidic Pump Empowered Sensitive Recognition and Active Transport of Hydrogen Peroxide in 1D Channels
Source: Adv Sci (Weinh). 2024 Nov 11;12(1):2408755. doi: 10.1002/advs.202408755 (PMC11714159; doi:10.1002/advs.202408755)
Supplement: Supplementary file 1 — Supporting Information [file ADVS-12-2408755-s003.docx]

Supporting Information

Hydrodynamic Fluidic Pump Empowered Sensitive Recognition and Active Transport of Hydrogen Peroxide in 1D Channels

*Shuya Liu^a^, Yongxian Guo^b^, Yanjun Gong^a^, Yanze Wei^a, c,^*, Qiongzheng Hu^b,^*, and Li Yu^a,^**

Shuya Liu, Prof. Yanjun Gong, Dr. Yanze Wei and Prof. Li Yu

Key Laboratory of Colloid and Interface Chemistry, Ministry of Education, Shandong University, Jinan 250100, China.

Dr. Yongxian Guo, and Prof. Qiongzheng Hu

Qilu University of Technology (Shandong Academy of Sciences), Shandong Analysis and Test Center, Jinan 250014, China.

Dr. Yanze Wei

State Key Laboratory of Biochemical Engineering, Institute of Process Engineering, Chinese Academy of Sciences, Beijing 100190 China

* E-mail address: yzwei@ipe.ac.cn; huqz@qlu.edu.cn; ylmlt@sdu.edu.cn;

**Table of Contents**

1. Experimental Section

2. Other Supporting Figures and Tables

3. References

1. **Experimental Section**

**1.1 Synthesis of D-A** **fluorophore 1 and fluorophore 2.**

**Scheme S1. Synthesis route of fluorophore 1 and fluorophore 2.**

**Methyl 4-(7-bromo-9,9-diethyl-9H-fluoren-2-yl)benzoate (5).** To a stirred solution of **3** (1.00 g, 5.5 mmol), **4** (2.50 g, 6.6 mmol), and tetrakis(triphenylphosphine)palladium (324 mg, 0.28 mmol) in deoxygenated 1,4-dioxane (40 mL), aqueous solution of potassium carbonate (8 mL, 2.0 M) was added. Then, the mixture was heated to 80 °C and stirred overnight under Ar. The solvent was evaporated under vacuum. The residue was poured into water (50 mL) and extracted with ethyl acetate (3 × 50 mL). The combined organic layer was washed with brine (satruated), dried over Na_2_SO_4_, and concentrated under vacuum. The residue was purified by column chromatography on the silica gel (petroleum: ethyl acetate = 20:1 as the eluent) to afford **5** (1.46 g, 3.4 mmol, 61% yield)

**Methyl 4-(9,9-diethyl-7-(4,4,5,5-tetramethyl-1,3,2-dioxaborolan-2-yl)-9H-fluoren-2-yl)benzoate (6).** A mixture of compound **5** (1.00 g, 2.3 mmol), potassium acetate (0.67 g, 6.9 mmol), bis(pinacolato)diboron (0.88 g, 3.5 mmol), and Pd(dppf)Cl_2_ (91 mg, 0.13 mmol) in deoxygenated 1,4-dioxane (30 mL) was stirred overnight at 80 °C under Ar. After removal of the solvent under vacuum, the residue was extracted with ethyl acetate (3 × 40 mL) and water (40 mL). The combined organic layers were washed with saturated NaCl solution, dried over Na_2_SO_4_, and concentrated under vacuum. The residue was purified by column chromatography (silica, petroleum: dichloromethane = 10:1) to afford compound **6** (1.06 g) in 96% yield.

**Methyl 4-(7-(benzo[c][1,2,5]thiadiazol-4-yl)-9,9-diethyl-9H-fluoren-2-yl)benzoate (2).** Compound **6** (1.06 g, 2.2 mmol), 4-bromobenzo[c][1,2,5]thiadiazole (0.47 g, 2.2 mmol), and tetrakis(triphenylphosphine)palladium (127 mg, 0.11 mmol) were added to a stirred deoxygenated 1,4-dioxane (20 mL) solution and an aqueous solution of potassium carbonate (2 M, 4 mL) was added into the above solution. Then, the mixture was stirred overnight at 80 °C under Ar. After removal of the solvent under vacuum, the residue was extracted with ethyl acetate (3 × 30 mL) and water (40 mL). The combined organic layers were dried over Na_2_SO_4_ and concentrated under vacuum. The residue was purified by column chromatography (silica, petroleum: dichloromethane = 5:1) to afford compound **2** (0.67 g) in 62% yield.

**4-(7-(benzo[c][1,2,5]thiadiazol-4-yl)-9,9-diethyl-9H-fluoren-2-yl)benzoic acid** (**1**) Compound **2** (500 mg, 1.0 mmol) was dissolved in 30 mL THF. Then, 8 mL 0.5 M water solution of KOH and tetrabutylammonium bromide (0.09 mmol, 29 mg) were added into the above solution. The mixture was refluxed for 10 h and then cooled to room temperature. HCl (1 M) was added to tune the pH value of the solution to 2-3. The resulting yellow powder was collected and dried in vacuum (yield 96%).


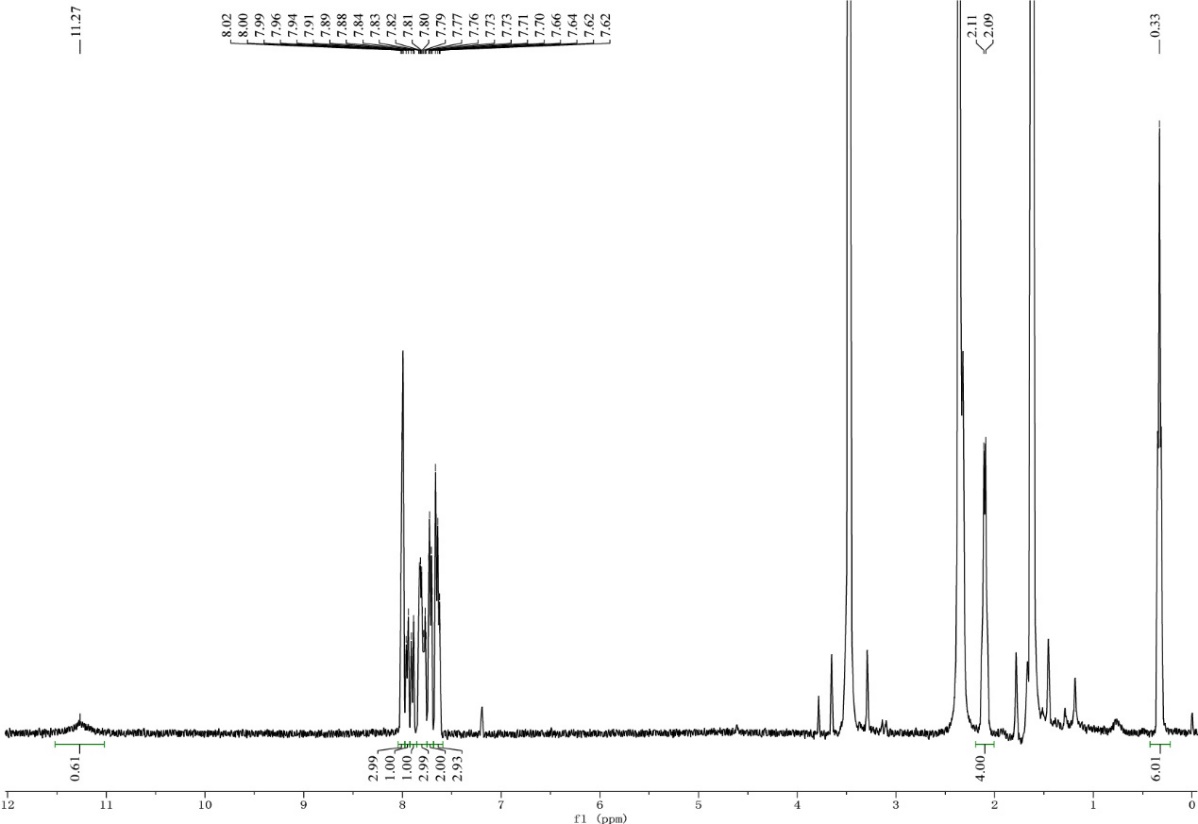


Figure S1. NMR data of fluorophore **1**.

Fluorophore **1**: ^1^H NMR (400 MHz, THF-d8) δ 11.27 (s, 1H), 8.02-7.99 (m, 3H), 7.96, 7.94 (d, J = 8 Hz, 1H), 7.91, 7.89 (d, J = 8 Hz, 1H), 7.84-7.76 (m, 3H), 7.73, 7.71 (d, J = 8 Hz, 2H), 7.66-7.62 (m, 3H), 2.13, 2.11, 2.09, 2.07 (q, J = 8 Hz, 4H), 0.33 (s, 6H).


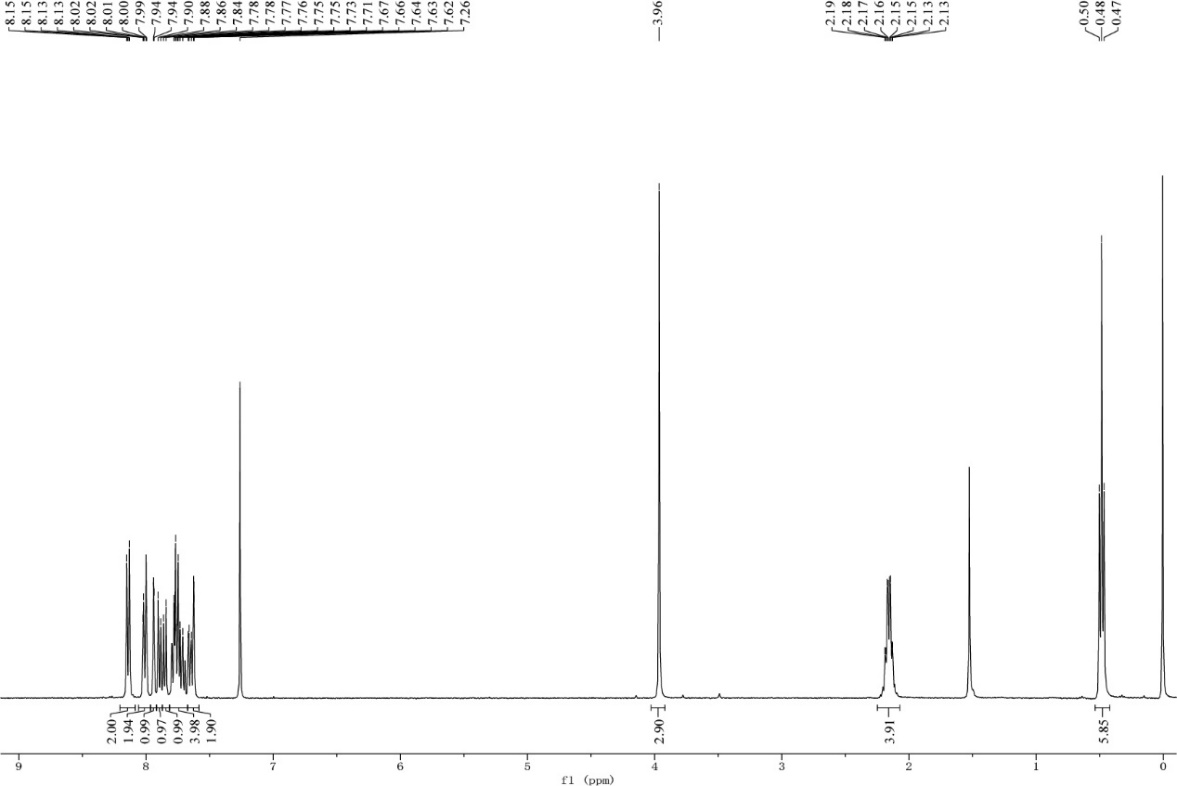


Figure S2. NMR data of fluorophore **2**.

Fluorophore **2**: ^1^H NMR (400 MHz, CDCl_3_): δ 8.15, 8.13 (d, J =8 Hz, 2H), 8.02,8.00 (d, J =8 Hz, 2H), 7.94 (s, 1H), 7.90,7.88 (d, J =8 Hz, 1H), 7.86,7.84 (d, J =8 Hz, 1H), 7.80-7.69 (m, 4H), 7.67, 7.65 (d, J =8 Hz, 1H), 7.63 (s, 1H), 3.96 (s,3H), 2.19-2.13 (m, 4H), 0.50, 0.48, 0.46 (t, J =8 Hz, 6H)

**1.2 Chemicals and Reagents**

Solvents including N,N-dimethylformamide (DMF), H_2_O_2_ (30 %, w/v), and Chloroform was purchased from Sinopharm Chemical Reagent Co., Ltd., China. Acetone, dichloromethane (DCM), methanol, ethanol, ethyl acetate and HCl were bought from Laiyang Kangde Chemical Co., Ltd. 1,3,6,8-tetrakis(p-benzoic acid)pyrene (H_4_TBAPy) was purchased from Jilin Chinese Academy of Sciences - Yanshen Technology Co., Ltd., China. ZrOCl_2_∙8H_2_O and Rhodamine B were purchased from Aladdin. Ionic liquid (IL) [C_4_mim][NTf_2_] was obtained from Leyan reagent (Shanghai, China). Super oxide dismutase (SOD) (15k units/mg) was purchased from Shanghai Yuanye Biological Technology Co., Ltd., (China). 5,5-Dimethyl-1-pyrroline *n*-oxide (DMPO) with 98% purity was bought from Beijing InnoChem Science & Technology Co., Ltd. Monodisperse polystyrene (PS) microspheres (excitations/emissions with 400 nm/450 nm) were purchased from 9-DingChem (Shanghai, China). Superoxide Anion Activity Content Assay Kit was bought from Shanghai Acmec Biochemical Co., Ltd. All reagents were purchased from commercial sources and used without further purification. Deionized water with conductivity of 0.055 μS/cm was purified by a Ulupure system.

**1.3 Synthesis of NU-1000, NU-1000-1, and NU-1000-1/IL composites.**

NU-1000 was synthesized following a procedure adopted from the literature.^1^ NU-1000-**1** was got through the SALI approach. NU-1000 (10 mg, 0.0045 mmol) was dispersed in 1 mL of DMF solution containing 5 mg/mL fluorophores **1** (0.01 mmol). After vertexing, the suspension was heated under 80 ℃ for 72 h, which allowed fluorophores **1** to reacted with NU-1000. The yellow NU-1000-**1** powder was washed with DMF (3×10 mL), acetone (3×10 ml), and methanol (3×10 mL), filtered, and dried under vacuum for 12 h. 1 mg of NU-1000-1 was dispersed in 500 μL of IL and vortexed for about 1 h to mix thoroughly. Then, the suspension was evacuated under vacuum condition for 48 h to ensure that IL was immersed within the channels of NU-1000-**1**. After that, excess IL was removed by filtration to obtain the resulting yellow powder named NU-1000-**1**/IL composites.

**1.4 Characterization.**

The fluorescence-mode optical microscopic images were obtained on an inverted fluorescence microscope (Olympus X71).

Scanning electron microscope (SEM) measurements were performed using a Hitachi S-8010 field-emission microscope. The SEM-EDS analyses were carried out the SEM coupled to a Bruker™ XFlash 6-60 SDD EDS Detector. The samples were analyzed at an accelerating voltage of 20 kV and at low vacuum (40 Pa). Samples were prepared by drop-casting the suspending crystals in solution onto a silica substrate followed by sputtering Pt on the surface (The sputtered Pt layer was ca. 3 nm).

Powder X-ray diffraction (PXRD) data were recorded on a Rigaku model SmartLab X-ray diffractometer using Cu Ka radiation.

Inductively coupled plasma optical emission spectroscopy (ICP-OES) was conducted on an Agilent 5110 ICP-OES spectrometer. Samples (2-3 mg) were digested in 3:1 v/v conc. H_2_SO_4_:H_2_O_2_ (30 wt% in H_2_O) and heated at 120 ℃ until the solution became clear and colorless and no further vapor was produced. An aliquot of this acidic solution was diluted to 5 vol% with ultrapure deionized H_2_O (resistivity >18 MΩ·cm, collected from a Milli-Q system) and analysed for Zr and S content as compared to standard solutions.

FTIR measurement was carried out using a Bruker Tensor II FTIR with the potassium bromide pellet technique.

Gas Uptake: N_2_ adsorption isotherm measurements were performed on a Micromeritics ASAP 2460-4MP analyzer at 77 K. Around 20 mg samples were used for each measurement. Prior to the measurement, all the samples were degassed under vacuum at 100 ℃ for 20 h using the internal turbopump.

UV-Visible absorption spectra were obtained on a PerkinElmer Lambda 35.

The fluorescence spectra and time-dependent fluorescence profiles were obtained by using an optical fiber connected to Ocean Optics USB4000 fluorometer to collect the emission and using a 385 nm LED lamp (Ocean Optics, total output power 3 mW) coupled with an optical fiber as the light source (0.053 mW/cm^2^).

Fluorescence quantum yield (QY) was measured by integrating a commercial spectrophotometer with an integrating sphere (IS).

**1.5 Photothermal Conversion Imaging Experiments.**

The 5 mg NU-1000-**1**/IL composites were spread on a glass. A 385 nm LED lamp (power 3 mW) was fixed 5 cm above the sample. A thermal infrared camera (Hikvision Digital Technology Co., Ltd. TPK20) was fixed on a diagonal 60 cm from the sample to capture the temperature evolution with the LED lamp on and off. The lens of the camera were adjusted so that the sample is in the center of the field of view. The adopted infrared camera has a working wavelength of 8.0-14.0 μm and a spatial resolution of 256×192 pix. The temperature sensitivity and measurement accuracy of the camera can reach ± 0.05 ℃ and ± 2 ℃, respectively.

**1.6 Sensing Experiments.**

The fluorescence sensing measurements of H_2_O_2_ vapor, H_2_O vapor, and other organic solvent vapors were performed using a home-built detector. NU-1000-**1** and NU-1000-**1**/IL composites were deposited on the quartz plate by casting 50 μL ethanol solution of the suspending NU-1000-**1** and NU-1000-**1**/IL composites. The plates with the sensing materials were dried by a blower and placed in a sensing chamber. Real-time fluorescence response was monitored by blowing 10 mL of the vapor of interest at a certain concentration into the chamber and the vapor was pumped into the chamber containing the pre-deposited sensing materials by an air pump (150 mL/min).

**1.7 Molecular Dynamics (MD) Simulations.**

The NU-1000/IL system containing 1000 IL pairs and 1 NU-1000 was built for Molecular Dynamic simulations. The system was allowed to equilibrate for 10 ns with a time step of 1 fs by using NVT simulations. Under temperature at 300 K, hidden walls were set on both sides of the x direction simulation box with the force equivalent to 1 atmospheric pressure. The NU-1000 was kept rigid to ensure that the atoms of the substrates were fixed during the simulation. The temperature of each confined case was maintained at 300 K by using a Nosé-Hoover thermostat with a damping factor of 100 fs. The Universal Force Field^2^ was used for NU-1000. The opls-aa force field^3^ was applied for cation and anion of ILs. The system was placed in periodic orthogonal boxes. And all the Molecular Dynamics simulations were performed by using LAMMPS software package.

**1.8 Finite Element Simulation.**

The three-dimensional geometry “CFD (computational fluid dynamics)” module was used to solve the flow velocity distribution in the channel. The simulation of natural convection heat transfer was first carried out using the natural convection interface in the CFD module. The laminar Navier-Stokes equations were used to solve the natural convection velocity field in the fluid region. Channels with lengths of 10 μm and diameters of 3 μm were constructed.

1. **Other Supporting Figures and Tables.**


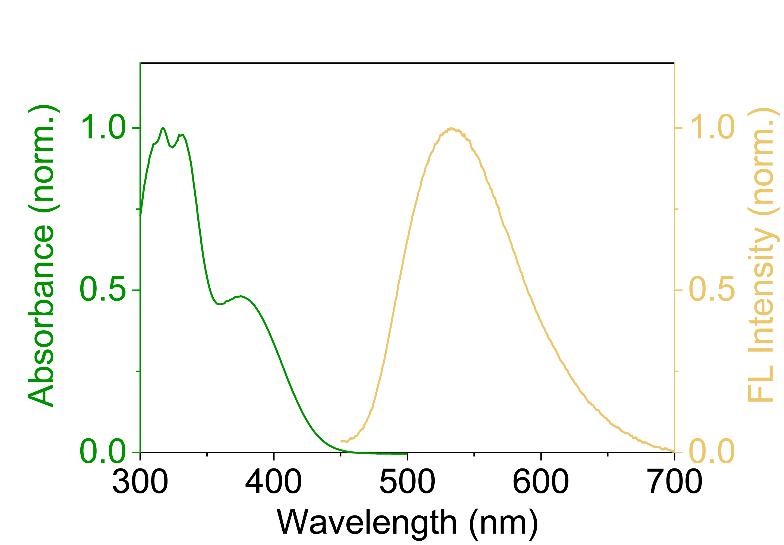


Figure S3. Normalized absorption and fluorescence spectra of **1** in DMF (5 μg/mL).


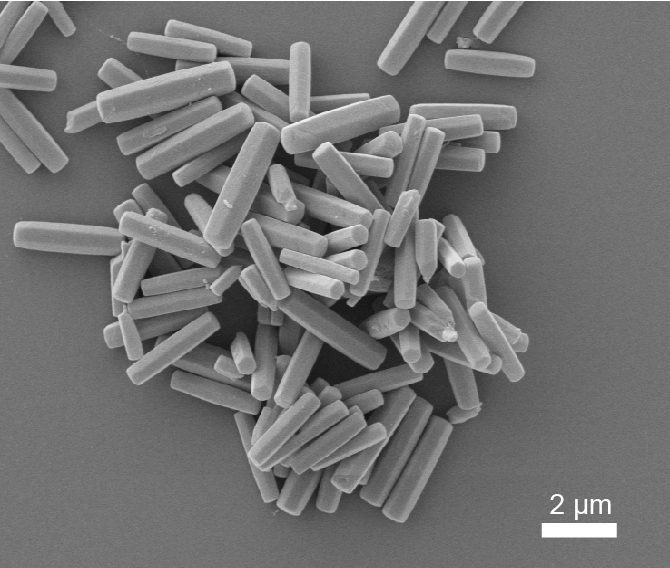


Figure S4. SEM image of NU-1000.


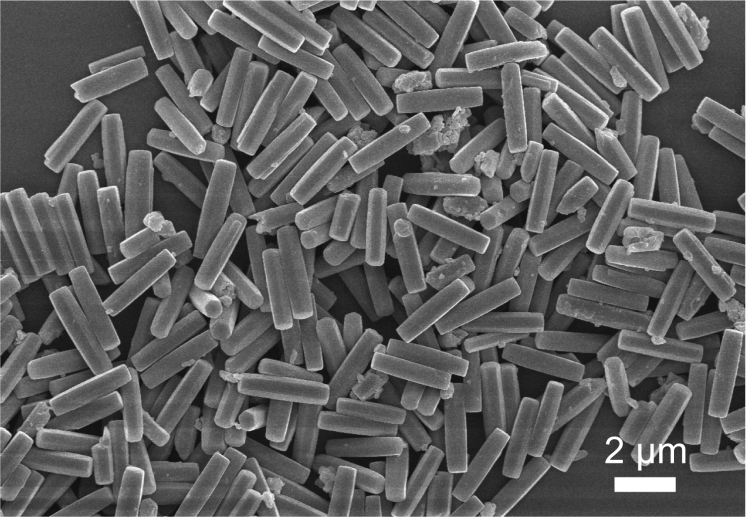


Figure S5. SEM image of NU-1000-**1.**


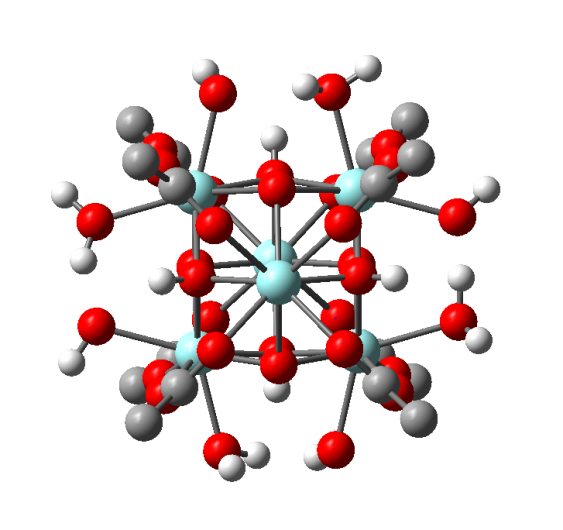


Figure S6. Molecular structure of the NU-1000 node. Color code: Zr (blue); O (red); C (grey); H (white).


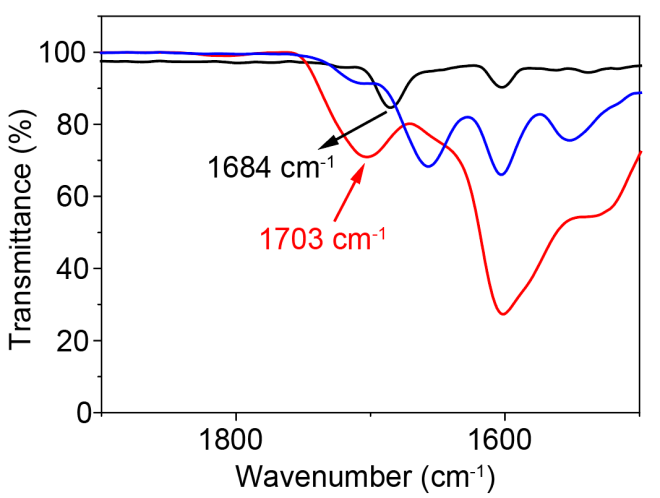


Figure S7. FTIR spectra of NU-1000 (blue), D-A fluorophore **1** (black), and NU-1000-**1** (red).


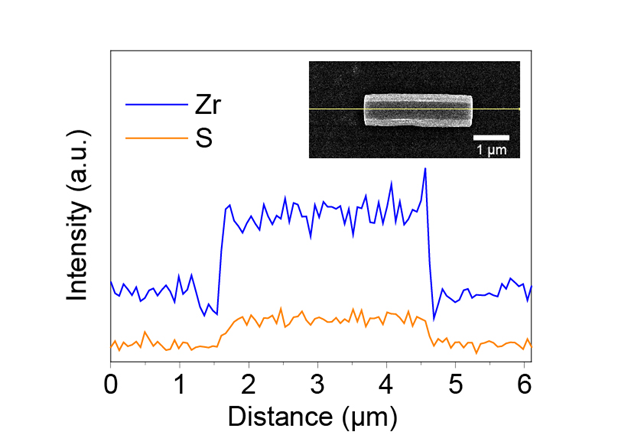


Figure S8. SEM/EDS line scan profiles of NU-1000-**1**.

Table S1. Molar ratio of Zr, S in NU-1000-**1** obtained by ICP.

|  | Sample mass *m*_0_ (g) | V (mL) | *C* (mg/L) | mM |
| --- | --- | --- | --- | --- |
| Zr | 0.0028 | 25 | 16.1 | 0.176 |
| S | 0.0028 | 25 | 0.95 | 0.029 |
| Zr/S=6.06 (Zr_6_ cluster/S ≈1) | | | | |


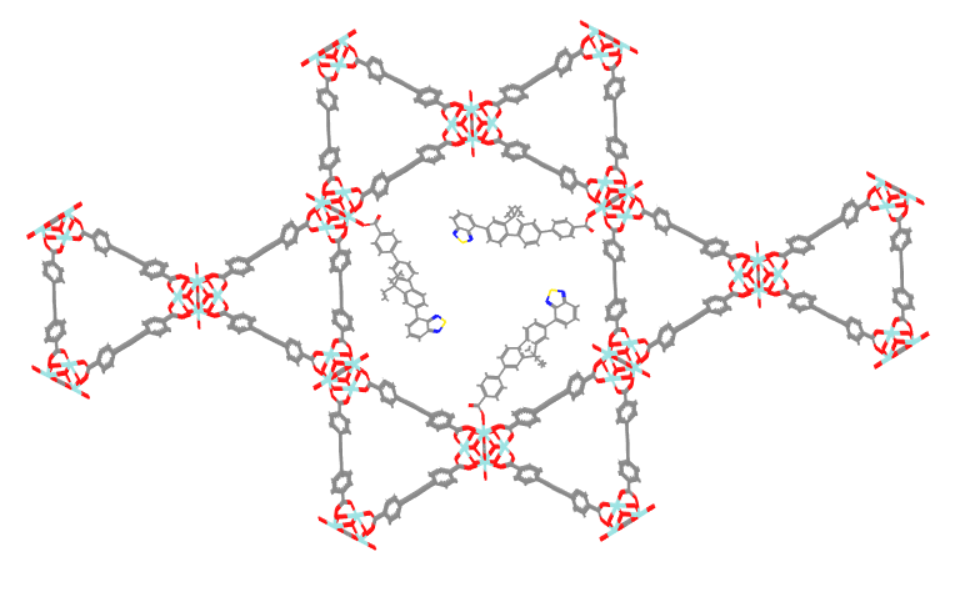


Figure S9. Structure of NU-1000-**1**.


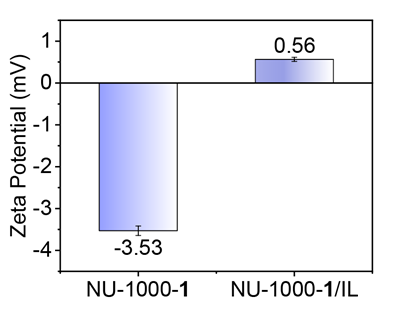


Figure S10. The zeta potential of NU-1000-**1** and NU-1000-**1**/IL.


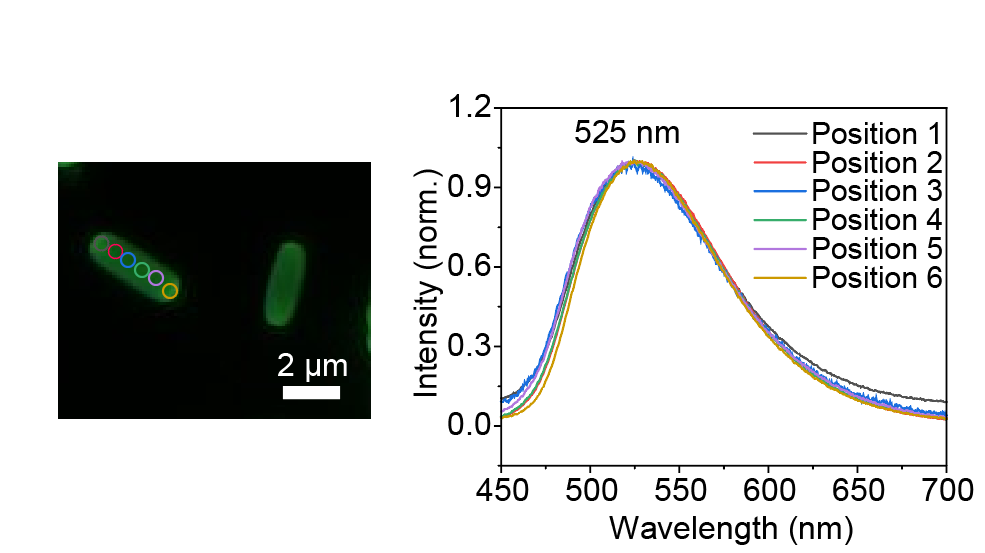


Figure S11. The fluorescence microscopy image, and spatially resolved fluorescence spectra of crystalline NU-1000-**1**/IL.

Figure S12. Normalized fluorescence spectra of fluorophore **2** in different solvents.


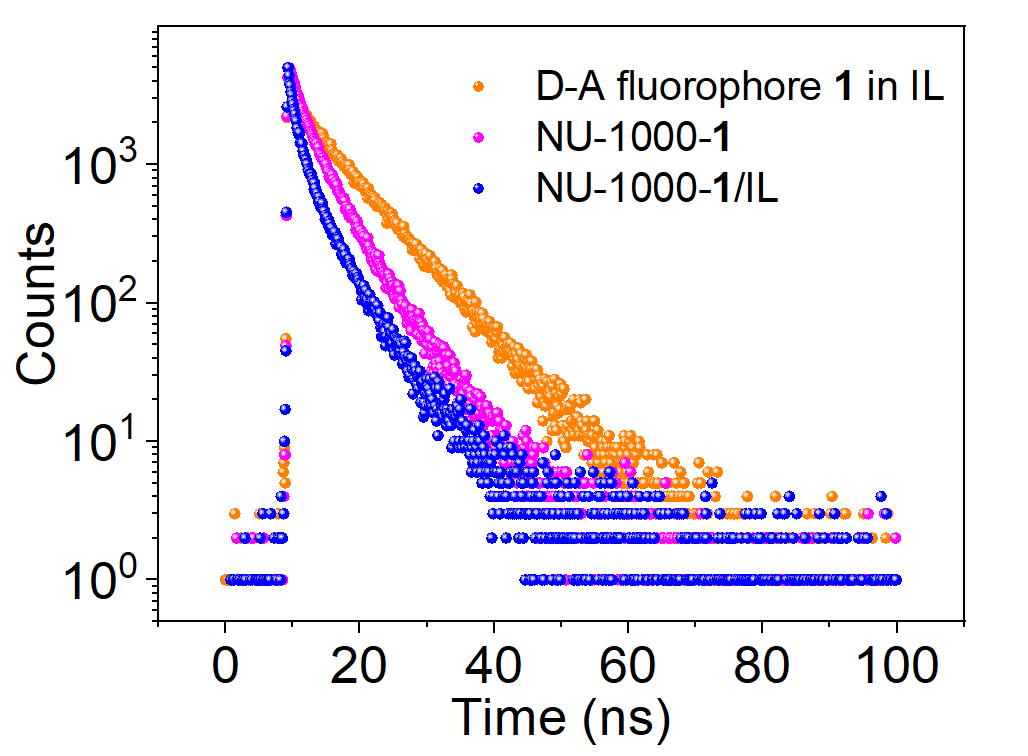


Figure S13. The fluorescence decay lifetimes for D-A fluorophore **1** in IL, NU-1000-**1**, and NU-1000-**1**/IL with 377 nm excited and emission at 550 nm.

Table S2. The fluorescence decay lifetimes of D-A fluorophore **1** in IL, NU-1000-**1**, and NU-1000-**1**/IL.

|  | τ_0_ (fraction) | τ_1_ (fraction) | | τ_2_ (fraction) | τ_avg_ | $\chi^{2}$ |
| --- | --- | --- | --- | --- | --- | --- |
| D-A fluorophore **1** in IL | 0.74 ns (10.88%) | 7.68 ns (89.12%) | / | | 6.92 ns | 1.06 |
| NU-1000-**1** | 2.02 ns (37.58%) | 5.61 ns (62.42%) | / | | 4.26 ns | 1.06 |
| NU-1000-**1**/IL | 0.65 ns (40.63%) | 2.34 ns (36.14%) | 6.26 ns (23.23%) | | 2.56 ns | 1.04 |


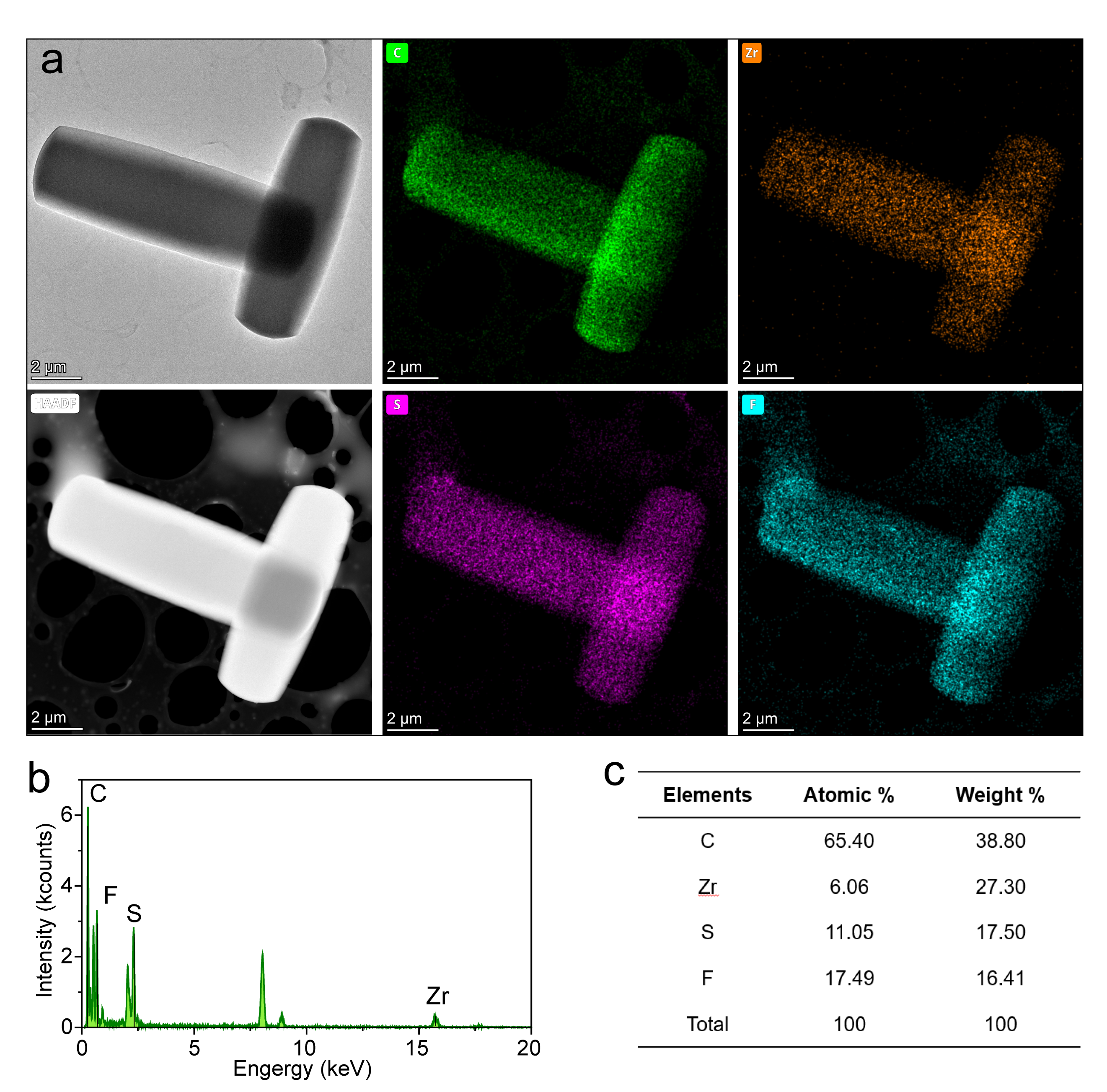


Figure S14. (a) TEM image, the high-angle annular dark-field scanning transmission electron microscopy (HAADF-STEM) images and EDX mapping of NU-1000-**1**/IL. (b-c) Qualitative-quantitative elemental mapping analysis of the C, Zr, S, F elements, obtained by EDX, for the synthesized NU-1000-**1**/IL.


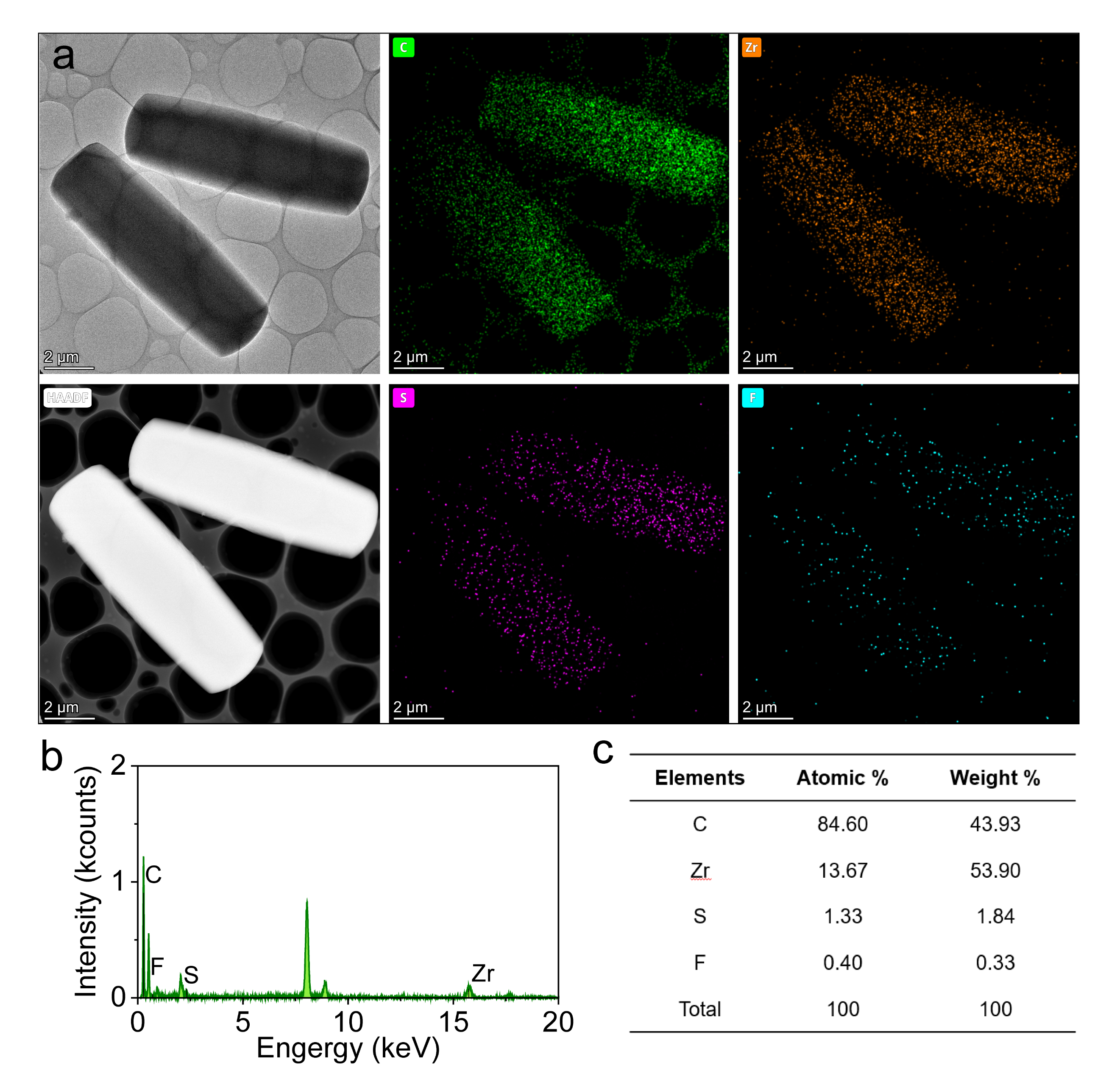


Figure S15. (a) TEM image, the high-angle annular dark-field scanning transmission electron microscopy (HAADF-STEM) images and EDX mapping of NU-1000-**1**. (b-c) Qualitative-quantitative elemental mapping analysis of the C, Zr, S, F elements, obtained by EDX, for the synthesized NU-1000-**1**.


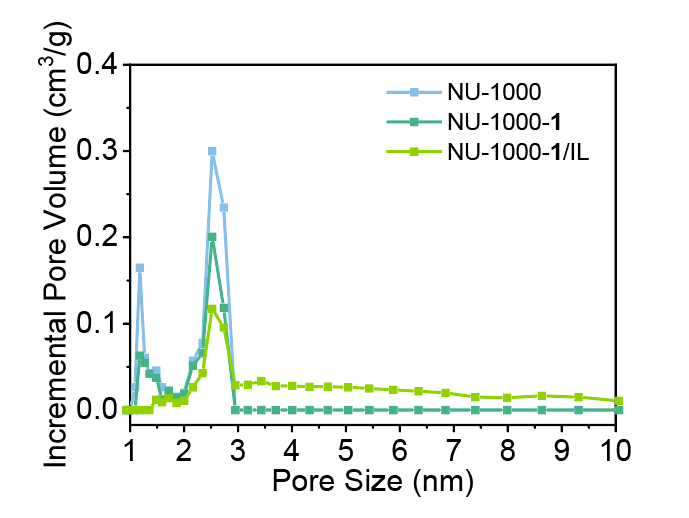


Figure S16. The pore size distributions of NU-1000, NU-1000-**1**, and NU-1000-**1**/IL composites, respectively.


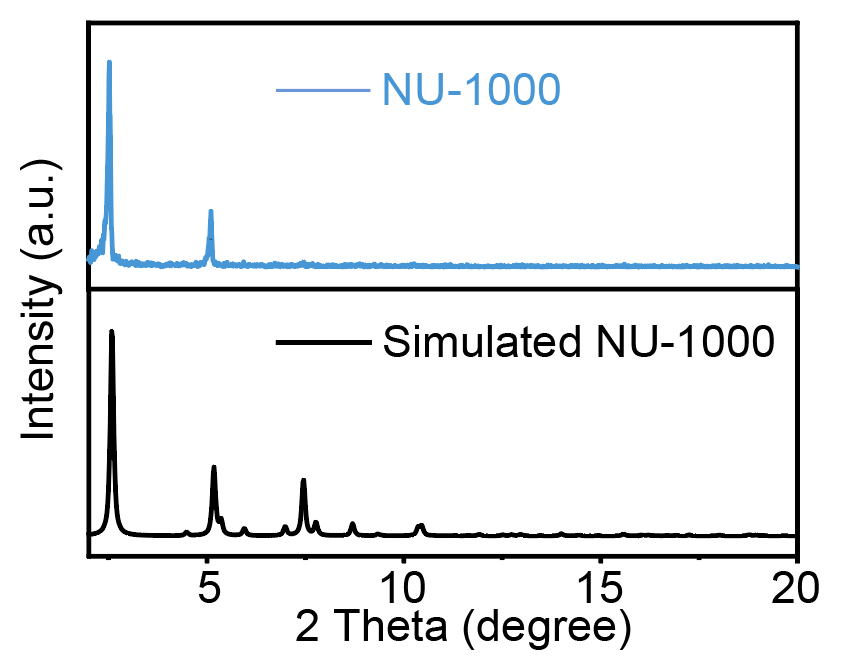


Figure S17. Experimental powder X-ray diffraction (PXRD) of NU-1000 crystalline together with simulated NU-1000.


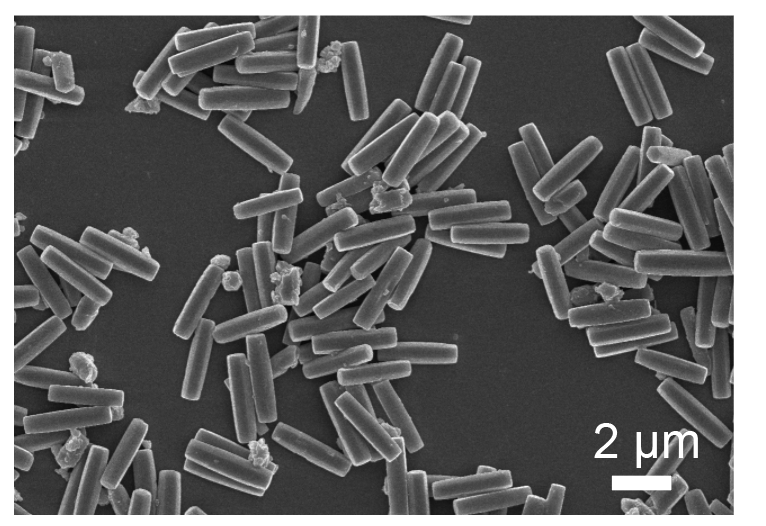


Figure S18. SEM image of NU-1000-**1/**IL.


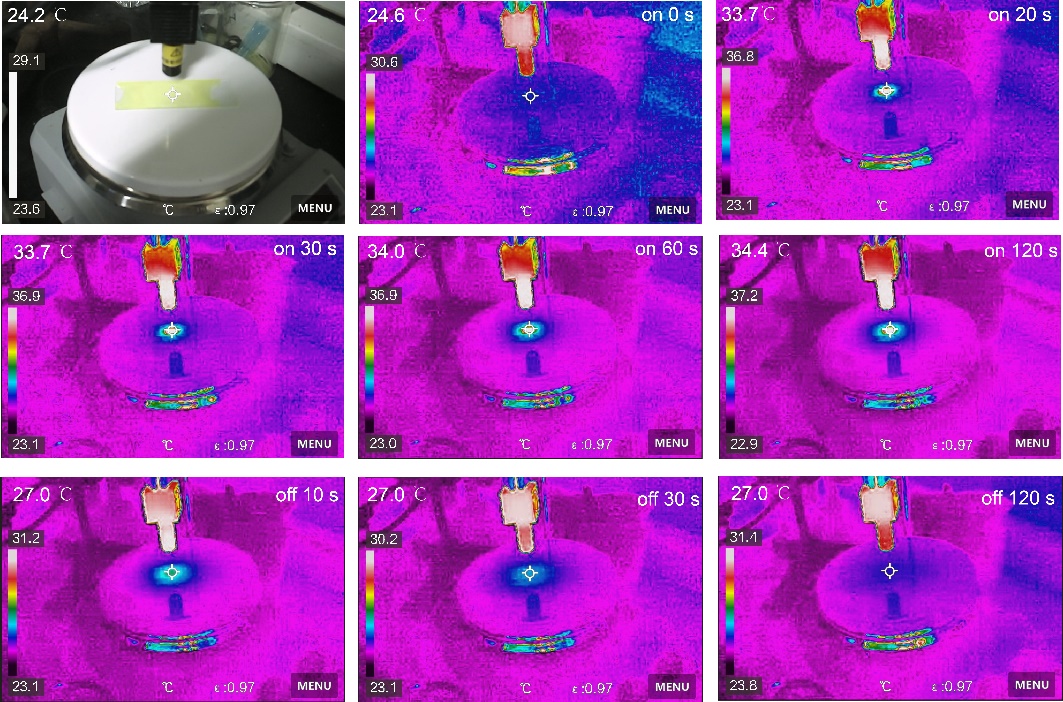


Figure S19. The thermal images of NU-1000**-1**/IL composites at different time.


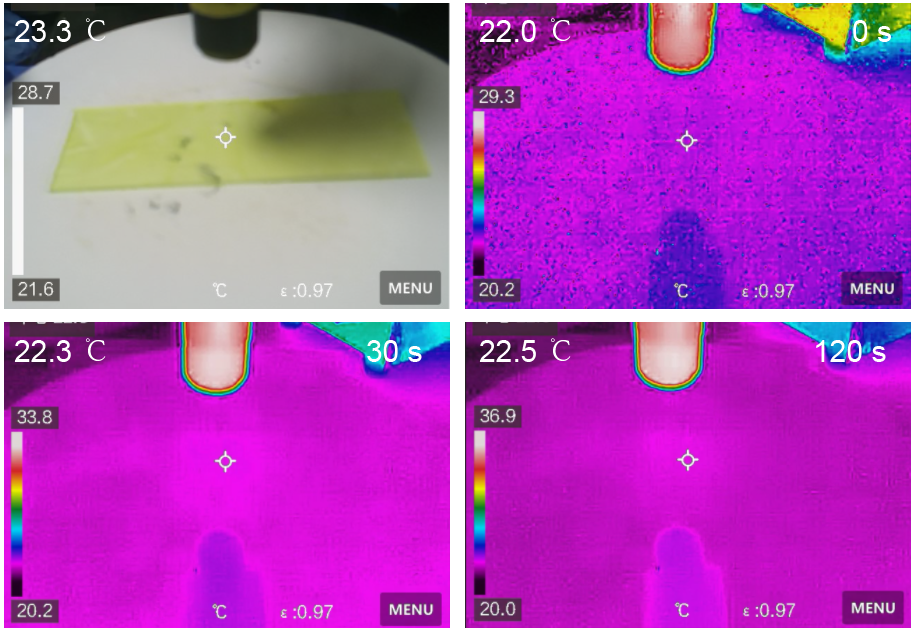


Figure S20. The thermal images of the control sample (NU-1000/IL) at different time.


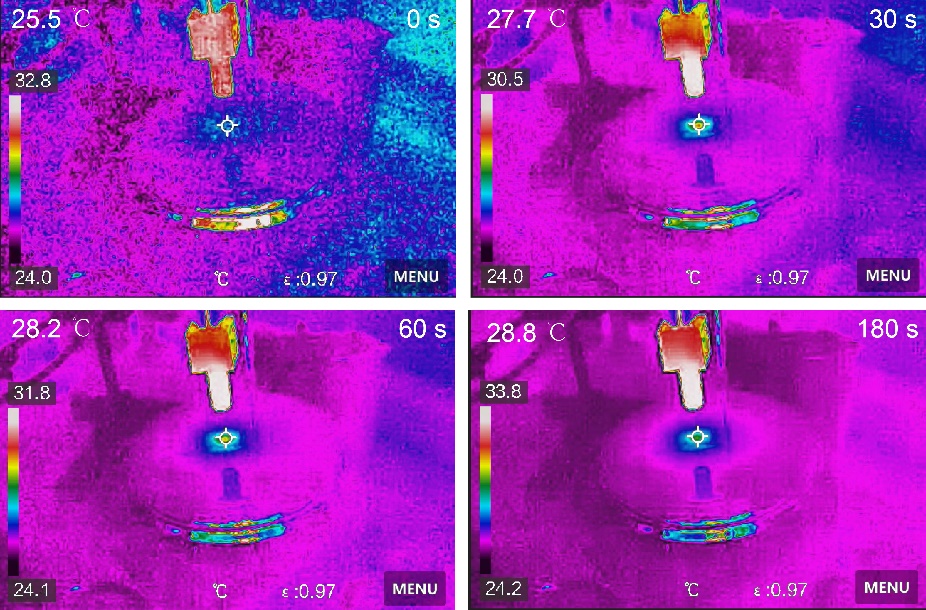


Figure S21. The thermal images of blank (only glass) at different time.


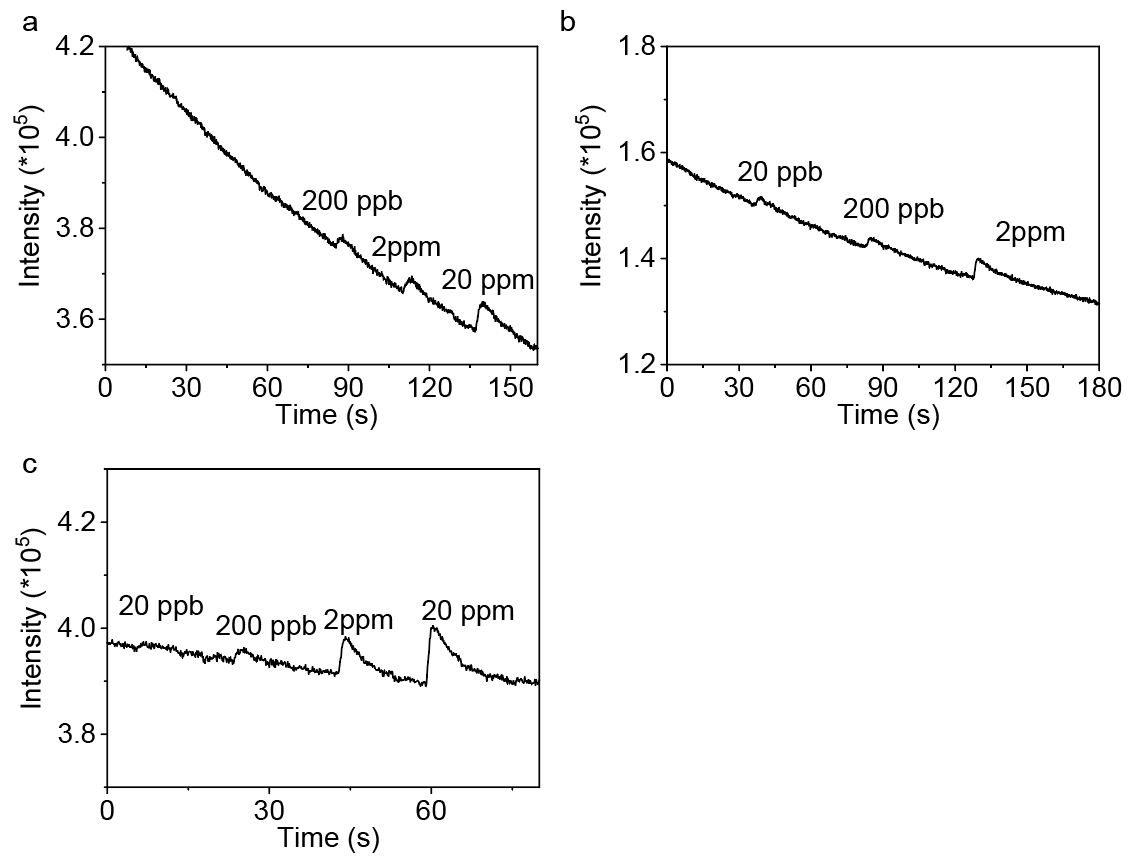


Figure S22. Time-dependent fluorescence profiles of (a) NU-1000/IL composites, and (b) NU-1000-**1**/IL composites post-modification in 2 mg/mL D-A fluorophore **1** when exposed to H_2_O_2_ vapor at various concentrations. We observed that the inclusion of D-A fluorophore **1** led to a tenfold increase in the detection limit for H_2_O_2_.


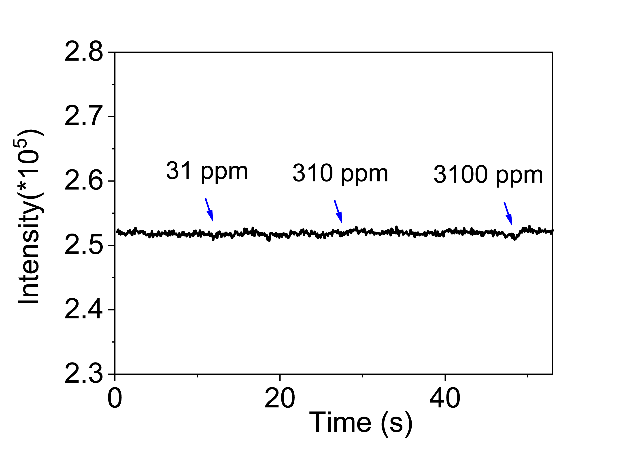


Figure S23. Fluorescent responses of the NU-1000-**1**/IL composites when exposed to H_2_O vapor at various concentrations.


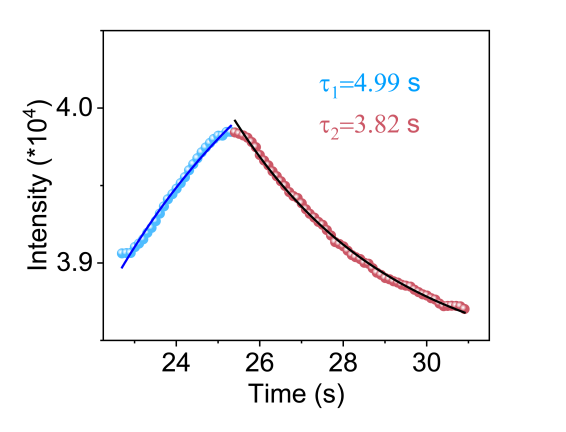


Figure S24. The response time (τ_1_) and recovery response time (τ_2_) of the NU-1000-**1**/IL composites when exposed to trace H_2_O_2_ vapor.


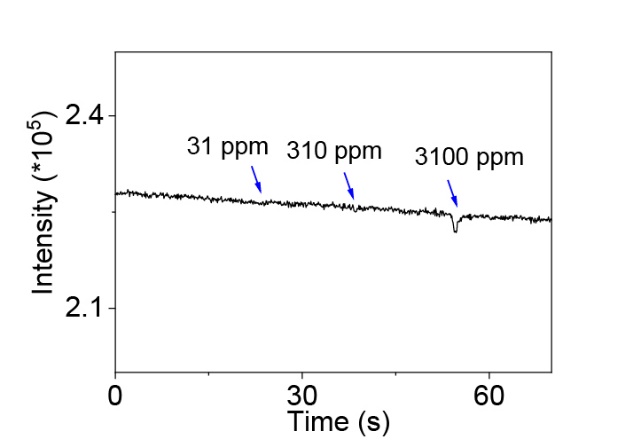


Figure S25. Fluorescent responses of the NU-1000-**1** composites to H_2_O vapor at various concentrations.


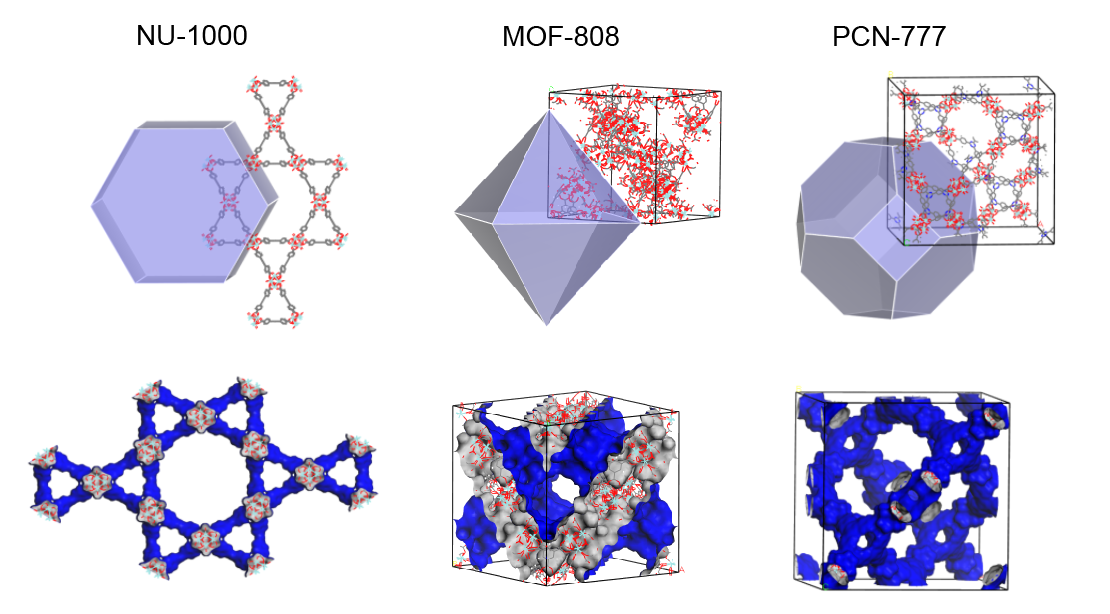


Figure S26. The crystal morphologies and pore structures of NU-1000, MOF-808, and PCN-777.


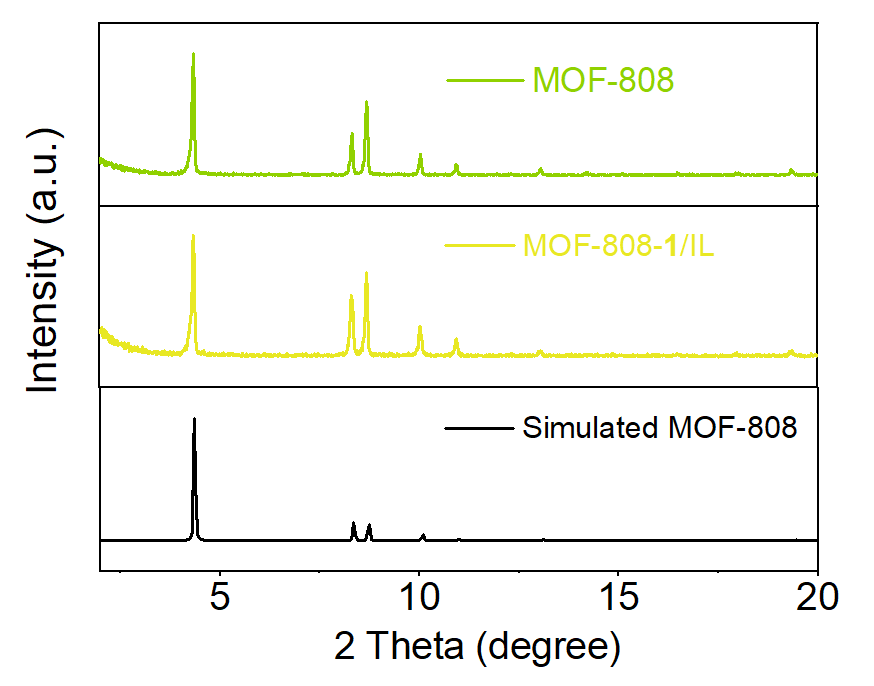


Figure S27. The experimental PXRD patterns of MOF-808, MOF-808-**1**/IL and simulated MOF-808.


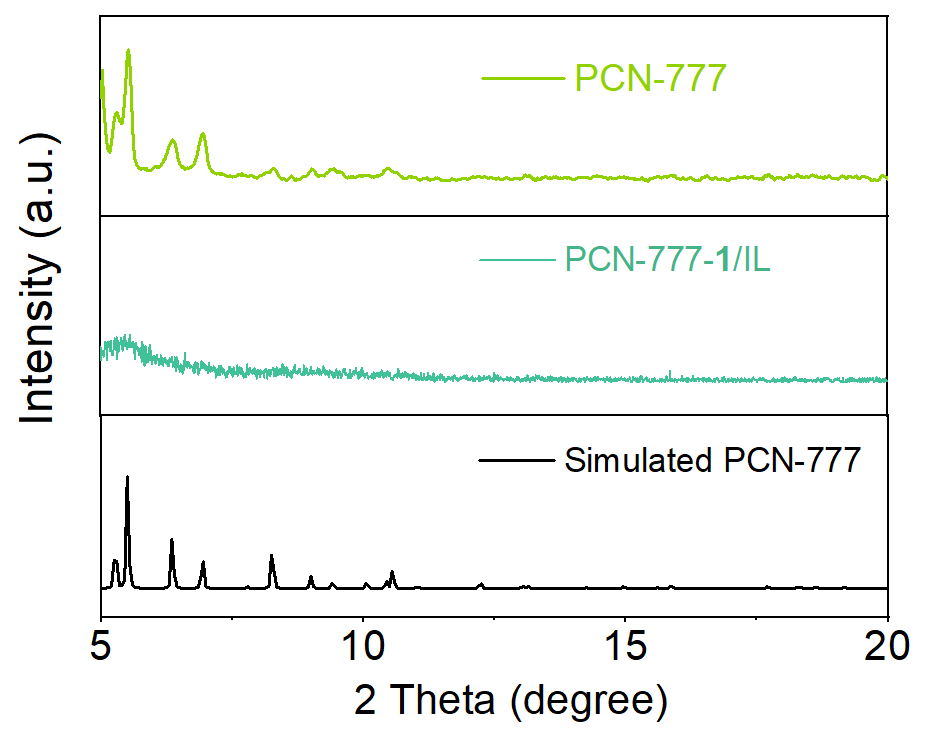


Figure S28. The experimental PXRD patterns of MOF-777, MOF-777-**1**/IL and simulated MOF-777.


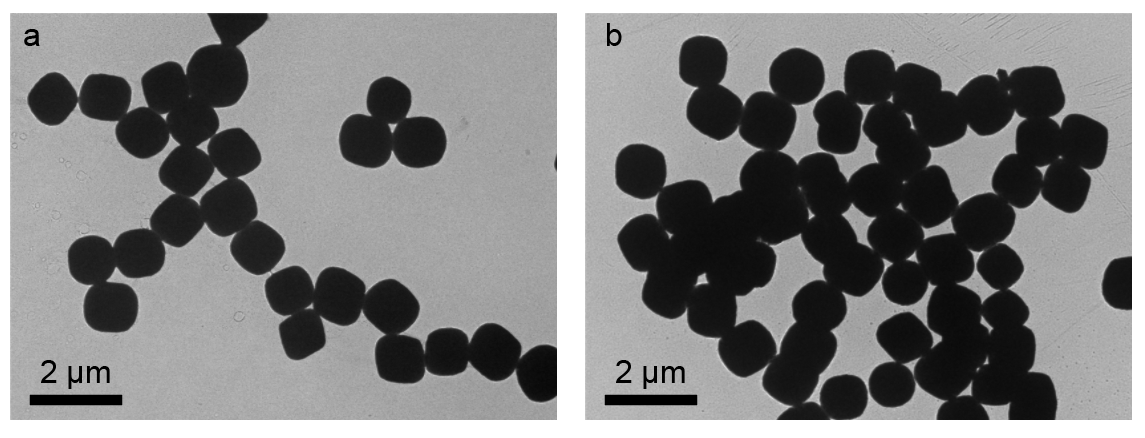


Figure S29. The TEM of MOF-808 and MOF-808-**1**/IL.


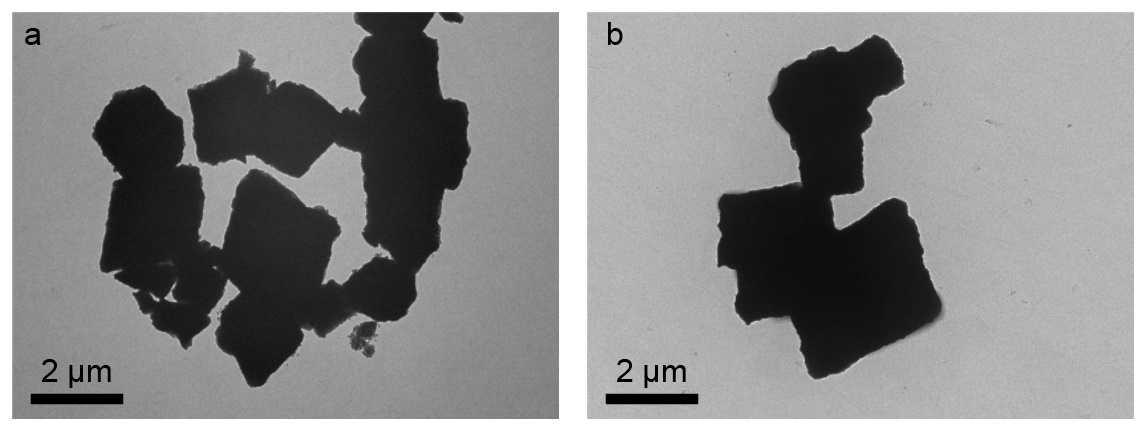


Figure S30. The TEM of PCN-777 and PCN-777-**1**/IL.


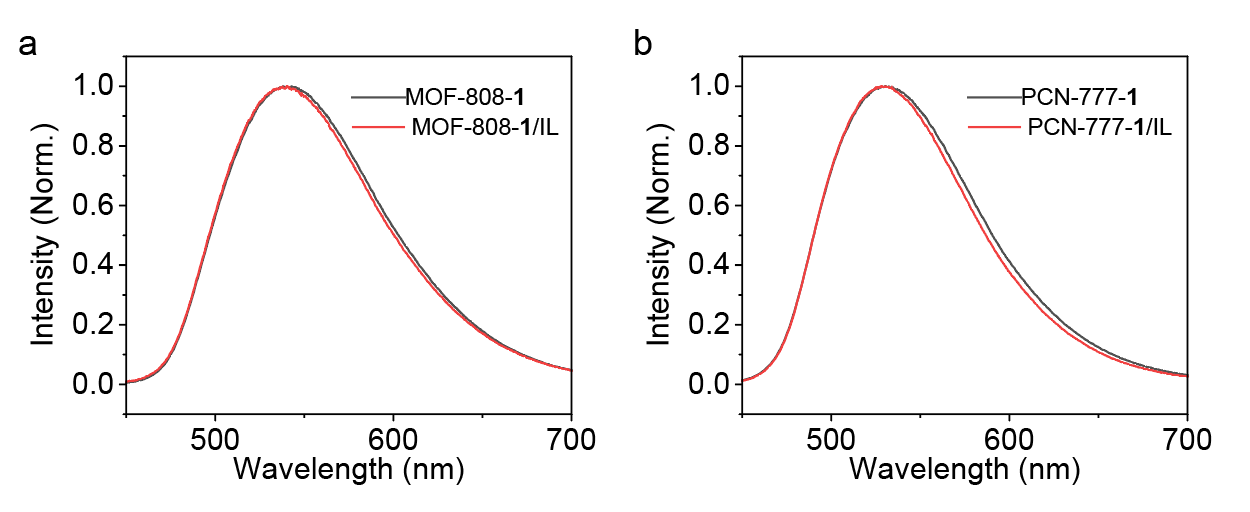


Figure S31. The fluorescence spectra of (a) MOF-808-**1** and MOF-808-**1**/IL, (b) PCN-777-**1** and PCN-777-**1**/IL, respectively.

The fluorescence spectra revealed that the main emission peaks for MOF-808-**1** and MOF-808-**1**/IL were essentially identical, located at 540 nm. This occurs maybe because the pore size is smaller than the length of D-A fluorophore **1** (19.2 Å), enabling the fluorophore to diffuse into and become entrapped within the pores, leading the ILs difficult enter into the pores. Similarly, the main emission peaks of PCN-777-**1** and PCN-777-**1**/IL, were consistent at 530 nm. That means D-A fluorophore **1** post-modification to PCN-777 for the 3.8 nm larger pores. However, the IL didn't surround the D-A fluorophore **1** for its non 1D-channels. The above experimental results illustrate that the IL in PCN-777-**1**/IL and MOF-808-**1**/IL is not around D-A fluorophore **1.**


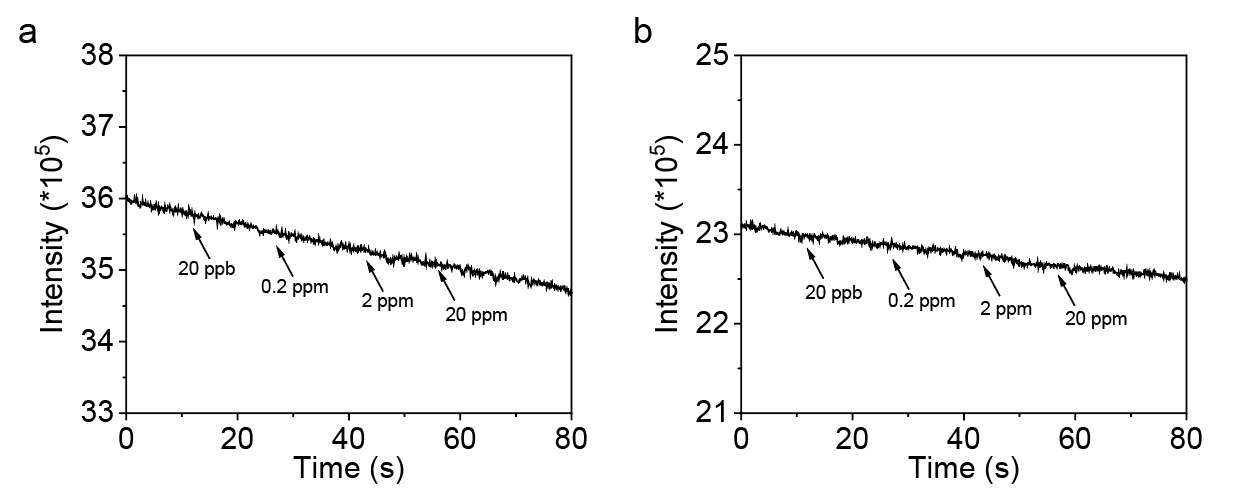


Figure S32. Time-dependent fluorescence profiles of (a) MOF-808-**1**/IL, and (b) PCN-777-**1**/IL when exposed to H_2_O_2_ vapor at various concentrations.


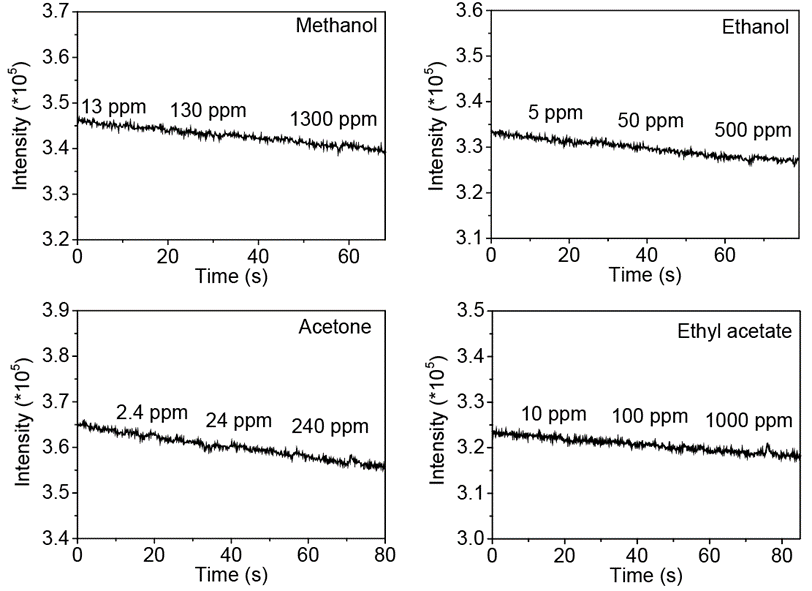


Figure S33. Fluorescent responses of the NU-1000-**1**/IL composites to other potential interfering vapors at various concentrations.

Table S3. Comparison of the detection of H_2_O_2_ vapor using different methods.

| Method | | Detection range | LOD | Selectivity | Response time | Reference |
| --- | --- | --- | --- | --- | --- | --- |
| Tri-Mode Visualization | colorimetric method | 0-34.4 ppm | 0.2 ppm | yes | 1 s | [4] |
|  | fluorescence | 0-16.4 ppm |  |  |  |  |
|  | upconversion luminescence (UCL) emission | 0-34.4 ppm |  |  |  |  |
| Fluorescence ratio | | 0-0.6 ppm | 7 ppb | yes | 10 min | [5] |
| Fluorescence increase | | 0.2-20 ppm | 0.2 ppm | yes | ~5 s | [6] |
| Electrochemical method | | 0.1-10 ppm | 2 ppb | yes | 12~40 s | [7] |
| Fluorescence increase | | 0.02-20 ppm | 0.02 ppm | yes | ~5 s | This work |


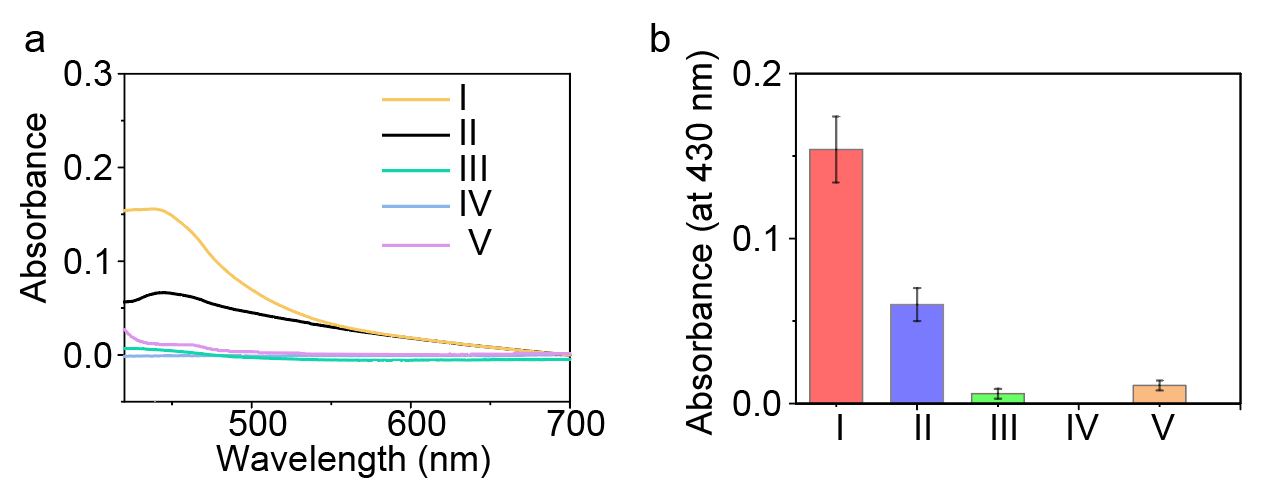


Figure S34. (a) UV-vis spectra and (b) effects of IL and reactive oxygen species scavengers on the oxidation efficiencies of TMB based on typical absorption at 430 nm (TMBox_2_). I: 30 μg/mL NU-1000**-1**, 0.4 mM [C_4_mim][NTf_2_], 1 mg/mL TMB, and 200 mM H_2_O_2_. II: 30 μg/mL NU-1000**-1**, 1 mg/mL TMB, and 200 mM H_2_O_2_. III: 0.4 mM [C_4_mim][NTf_2_], 1 mg/mL TMB, and 200 mM H_2_O_2_. IV: 1 mg/mL TMB, and 200 mM H_2_O_2_. V: 30 μg/mL NU-1000**-1**, 0.4 mM [C_4_mim][NTf_2_], 1 mg/mL TMB, 200 mM H_2_O_2_ and 3000 U/mL SOD.


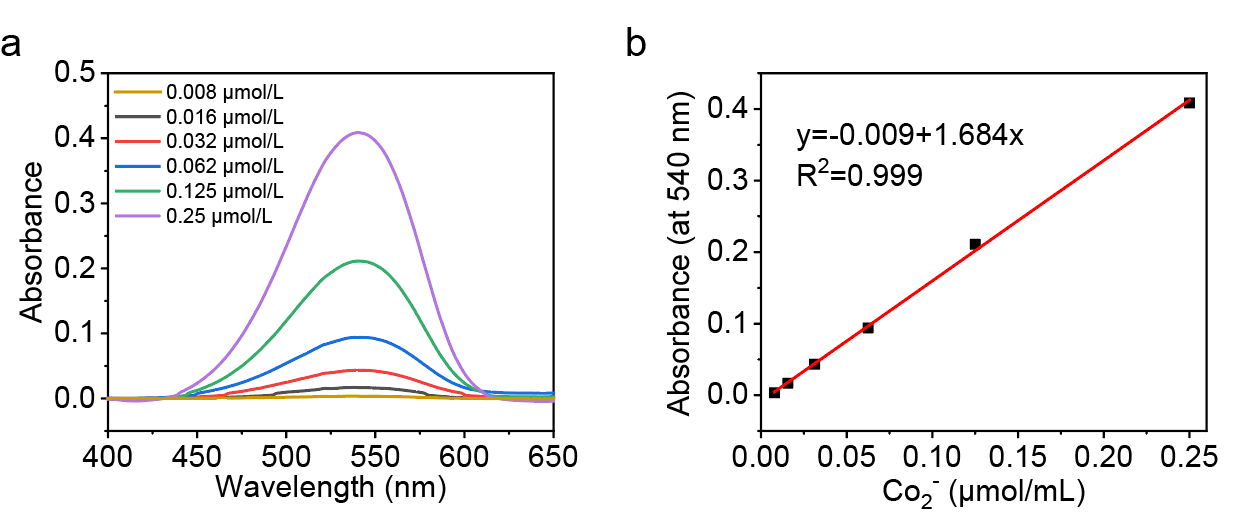


Figure S35. (a) UV-vis spectra of different concentrations of superoxide anions using the hydroxylamine hydrochloride colorimetric method. (b) Fitted standard curve of experimental absorbance at 540 nm for different concentrations of superoxide anions.


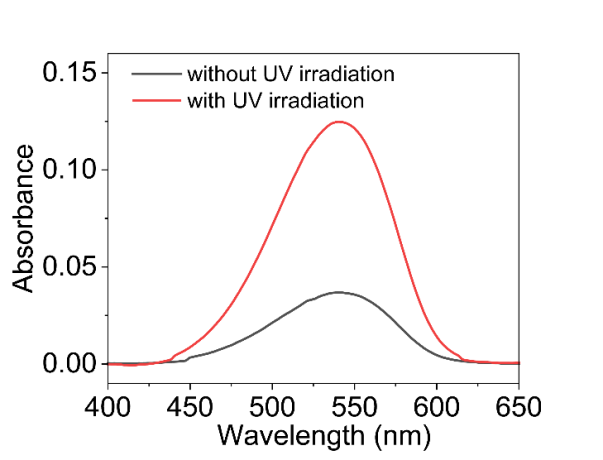


Figure S36. UV-vis absorption spectra of water containing H_2_O_2_-loaded NU-1000-**1**/IL in different conditions.

Table S4. Concentrations of superoxide anions obtained from the standard curve.

| Condition | Absorbance | C_·O2_^-^ (μmol·L^-1^) |
| --- | --- | --- |
| without UV irradiation | 0.037 | 0.027 |
| with UV irradiation | 0.125 | 0.080 |

**3. References**

(1) Wang, T. C.; Vermeulen, N. A.; Kim, I. S.; Martinson, A. B.; Stoddart, J. F.; Hupp, J. T.; Farha, O. K. Scalable Synthesis and Post-modification of a Mesoporous Metal-organic Framework Called NU-1000. *Nat. Protoc.* **2016**, *11*, 149-162.

(2) Boyd, P. G.; Moosavi, S. M.; Witman, M.; Smit, B. Force-Field Prediction of Materials Properties in Metal-Organic Frameworks. *J. Phys. Chem. Lett.* **2017**, *8*, 357-363.

(3) Watkins, E. K.; Jorgensen, W. L. Perfluoroalkanes:  Conformational Analysis and Liquid-State Properties from ab Initio and Monte Carlo Calculations. *J. Phys. Chem. A* **2001**, *105* (16), 4118-4125.

(4) Y. Feng, D. Lei, B. Zu, J. Li, Y. Li, X. Dou, A Self-Accelerating Naphthalimide-Based Probe Coupled with Upconversion Nanoparticles for Ultra-Accurate Tri-Mode Visualization of Hydrogen Peroxide. *Adv. Sci.* **2024**, *11*, 2309182.

(5) X. An, Y. Liu, Y. Sun, X. Zhang, Y. Liu, Y. Tao, L. Guo, X. Jiang, M. Gao, Portable multifunctional sensing platform for ratiometric H_2_O_2_ detection and photodynamic anti-bacteria using an AIE-featured electrospinning film. *Chem. Eng. J.* **2024**, *487*, 150675.

(6) X. Yu, Y. Gong, W. Xiong, M. Li, J. Zhao, Y. Che, Turn-on Fluorescent Detection of Hydrogen Peroxide and Triacetone Triperoxide via Enhancing Interfacial Interactions of a Blended System. *Anal. Chem.* **2019**, *91*, 6967-6970.

(7) J.-S. Lee, D.-W. Jeong, Y. T. Byun, Porphyrin nanofiber/single-walled carbon nanotube nanocomposite-based sensors for monitoring hydrogen peroxide vapor. *Sens. Actuators, B: Chem.* **2020**, *306*, 127518.
